# Drug synergy discovery of tavaborole and aminoglycosides against *Escherichia coli* using high throughput screening

**DOI:** 10.1186/s13568-022-01488-6

**Published:** 2022-12-01

**Authors:** Shasha Liu, Pengfei She, Zehao Li, Yimin Li, Linhui Li, Yifan Yang, Linying Zhou, Yong Wu

**Affiliations:** 1 Department of Laboratory Medicine, The Third Xiangya Hospital, Central South University, Changsha, 410000 Hunan China; 2 Department of Laboratory Medicine, The Affiliated Changsha Hospital of Xiangya School of Medicine, Central South University, Changsha, 410000 Hunan China

**Keywords:** Drug repurposing, Microbial proteomics, Benzoxaborole, Drug resistance, Uropathogenic *Escherichia col*

## Abstract

High incidences of urinary tract infection (UTI) of aminoglycosides-resistant *E.coli* causes a severe burden for public health. A new therapeutic strategy to ease this crisis is to repurpose non-antibacterial compounds to increase aminoglycosides sensibility against multidrug resistant *E.coli* pathogens. Based on high throughput screening technology, we profile the antimicrobial activity of tavaborole, a first antifungal benzoxaborole drug for onychomycosis treatment, and investigate the synergistic interaction between tavaborole and aminoglycosides, especially tobramycin and amikacin. Most importantly, by resistance accumulation assay, we found that, tavaborole not only slowed resistance occurrence of aminoglycosides, but also reduced invasiveness of *E.coli* in combination with tobramycin. Mechanistic studies preliminary explored that tavaborole and aminoglycosides lead to mistranslation, but would be still necessary to investigate more details for further research. In addition, tavaborole exhibited low systematic toxicity in vitro and in vivo, and enhanced aminoglycoside bactericidal activity in mice peritonitis model. Collectively, these results suggest the potential of tavaborole as a novel aminoglycosides adjuvant to tackle the clinically relevant drug resistant *E. coli* and encourages us to discover more benzoxaborole analogues for circumvention of recalcitrant infections.

**Supplementary Information:**

The online version contains supplementary material available at 10.1186/s13568-022-01488-6.

## Key points

- Benzoxaborole Compound Tavaborole was discovered against E. coli by using HTS.
- Tavaborole showed synergistic efficacy with aminoglycosides in vitro and in vivo.
- The main mechanism of synergy was promoting protein mistranslation mutually.

**Supplementary Information:**

The online version contains supplementary material available at 10.1186/s13568-022-01488-6.

## Introduction

*Escherichia coli* is a multitudinous collection with varied identities that is one of the most serious pathogens and the most widespread mammalian gastrointestinal commensals (Riley 2020). *E.coli* is the most common Gram-negative bacteria associated with blood-stream infections (BSI) worldwide and it is the primary cause of community-acquired urinary tract infections (UTIs), accounting for 70-95% of community-acquired UTI and 50% of hospital-acquired UTIs (Riley 2014; Zeng et al. 2021). In the last several decades, aminoglycosides antibiotics (AGAs) have shown remarkable clinical performance as first-line treatments for UTIs. It’s universally acknowledged that AGAs are recognized as an important broad-spectrum antibiotic that interferes with polyprotein synthesis by binding to the A-site of the 30 S ribosomal subunit (16 S rRNA) (Kulik et al. 2018). However, with the surge and dissemination of resistance, the usage of AGAs was declining by 41% (Goodlet et al. 2019; Ramirez and Tolmasky 2010). According to statistics, nearly 150 million UTIs occur worldwide each year, generating over $6 billion in medical expenditure (Stamm and Norrby 2001). Furthermore, based upon the European Centre for Disease Prevention and Control (ECDC), the prevalence of aminoglycosides-resistant *E. coli* bacteria in European countries ranged from 3.7 to 23.9% (Allocati et al. 2013). As a result, novel therapy strategies are required to tackle this ubiquitous clinical problem. One option is to design novel medicines with higher RNA binding affinity, better antibacterial activity, and more resistant-resistance (Busscher et al. 2005). Given that new drug discovery has several weaknesses, such as high attrition, unnecessary costs, low pace and so on, medication repositioning has emerged as a viable alternative option for obstinate bacterial infections or antibiotic sensitivity recovery (Pushpakom et al. 2019).

**Fig. 1:**
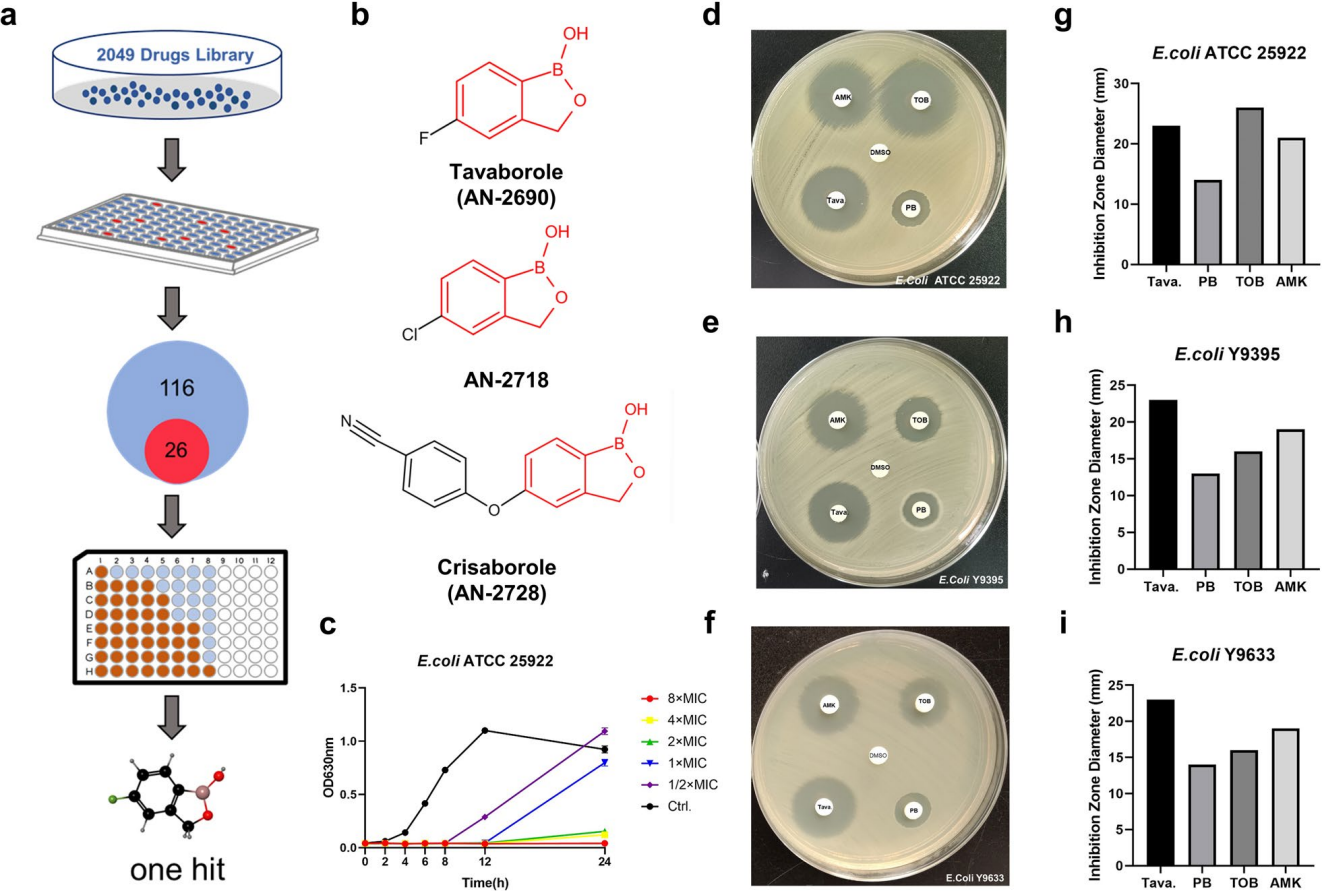
Tavaborole shows potent antibacterial activity against *E. coli*. **a** The scheme of high throughput screen for repurposing compounds. **b** Chemical structures of tavaborole and other benzoxaborole compounds. The structure of the benzene ring was highlighted in red. **c** *E. coli* ATCC 25,922 growth curves over 24 h in various tavaborole concentrations. The inhibition area of tobramycin (TOB), amikacin (AMK), polymyxin B (PMB), and tavaborole (Tava) against *E. coli* ATCC 25,922, Y9395, and Y9633 respectively was observed (**d**-**f**) and measured (**g**-**i**). One-way ANOVAs were performed

Herein, through high throughput screening (Ducret et al. 2016), we aimed to uncover antibacterials that disrupts bacterial membrane to aid antibiotics uptakes or inhibits protein translation process resulting in amplify growth defects. Therefore, we designed a protocol (Fig. 1A) in virtue of rifampicin, a bacterial RNA polymerase inhibitor with high molecular weight, and we ultimately chose tavaborole for further investigation (Campbell et al. 2001). Tavaborole (AN2690) is the first benzoxaborole antifungal drug approved by the Food and Drug Administration (FDA) in 2014 for onychomycosis treatment (Markham 2014). According to recent researches, tavaborole limits cell growth by directly inactivating leucyl-tRNA synthetase (LeuRS) and inhibiting protein synthesis (Melnikov et al. 2020). LeuRS is constituted by the synthesis site, where leucine is allowed to undergo programmed insertion for the creation of leucyl-tRNA^Leu^, and the editing site, where nonhomologous isoleucine and norvaline are cleared for proper quality control (Cvetesic et al. 2014). Tavaborole creates a covalent bond with tRNA^Leu^ and prevents its dissociation from LeuRS, generating protein synthesis inhibition and cell growth restriction (Melnikov et al. 2020). Apart from tavaborole, additional novel benzoxaborole compounds show antimicrobial and anticancer properties and have tremendous potential for further research. AN11527, for example, is an effective growth antagonist for *Mycobacterium TB* and *E. coli* (Mandal and Parish 2021). In ovarian melanoma cells, 3-morpholine-5-fluorobenzoxaborole causes a strong cell cycle halt (Psurski et al. 2019). AN3661 is a potent antimalarial benzoxaborole that targets the polyadenylation specificity factor homologue in Plasmodium falciparum (Sonoiki et al. 2017).

Synergistic therapy, as we all know, is not readily available, but it is a viable direction with many benefits, including lesser consumption, fewer side effects, and a slower evolution of resistance (Copp et al. 2020). Surprisingly, we discovered that tavaborole had outstanding synergistic activity with aminoglycosides, which is more useful in healthcare treatment than a single antibiotic property.

In our research, we hope to investigate the synergistic effects of tavaborole and AGAs against *E. coli*, as well as the role of tavaborole in AGAs resistance. Mechanistic studies revealed that the absorption and mis-regulation of AGAs was likely aided by tavaborole, which led to RNA mistranslation. Finally, the synergistic effectiveness was validated in mouse models with multidrug-resistant (MDR) bacterial infection. Our findings suggest that tavaborole could be used as a novel aminoglycoside adjuvant in clinical settings to combat multidrug-resistant *E. coli*.

## Materials and methods

### Strains, reagents and media

*Staphylococcus aureus* ATCC 43,300, Newman, RJ-2 and MDR *Escherichia coli* Y9633, Y9592, Y9395 and Y0064 were amicably provided by Min Li (Shanghai Jiaotong University, Shanghai, China). *Staphylococcus epidermidis* RP62A were obtained from Qu Di (Shanghai Medical College of Fudan University, Shanghai, China). In addition, *S. aureus* SAJ-1 and other *E. coli* clinical isolates were collected in the Third Xiangya Hospital of Central South University (Changsha, China). *Klebsiella pneumoniae* ATCC 700,603, and *Escherichia coli* ATCC 25,922 were provided by Juncai Luo (Tiandiren Biotech). *Acinetobacter baumannii* ATCC 19,606 was purchased from BeNa Culture Collection (BNCC, Beijing, China). *Pseudomonas aeruginosa* PAO1 came from Mingqiang Qiao research group (Nankai University). Gram-positive (G^+^) and Gram-negative (G^−^) strains were cultured in trypsin soybean broth (TSB, Solarbio, Shanghai, China) and Luria–Bertani (LB) broth medium (Solarbio), respectively. All bacteria were propagated at 37 °C with shaking at 180–200 rpm. Tavaborole and other chemicals were purchased from MedChem Express (New Jersey, the United States) or Sigma Aladdin (Shanghai, China).

### High throughput screen (HTS) of Antimicrobial Compounds

A drug library was screened for identification of promising agents against G- bacteria. This library contains ready-made 2049 FDA-approved and pharmacopeial chemicals (arranged in 96-microwell plates), including antimicrobials and other drugs with a wide range of chemical configurations and therapeutic applications. These compounds were stored at − 20  °C in 10 mM dimethyl sulfoxide (DMSO) with 30 µl of primary stock solutions. G^−^strains were grown to the mid-logarithmic phase and 0.5 McFarland (McF) turbidity standard bacterial suspension was prepared in LB culture medium with 1/2 × MIC rifampicin (RFP) as the base solution. For each well, 100 µg/ml individual chemical was added after transferring 99 µl base solution into clean 96-well plates. The culture condition is at 37 °C with 5% CO_2_ humid surroundings. The OD_630_ was measured after 16-hour incubation at 37 °C. All chemicals were repeated three times. Excluded antimicrobials, hit compounds were rescreened using a checkboard assay with RFP, and one hit was selected for further investigation (Domenech et al. 2020).

### Antimicrobial susceptibility testing (AST)

The protocol was designed by microdilution method to evaluate the antibacterial activity of tavaborole and other benzoxaborole compounds in vitro, according to the guidelines from Clinical & Laboratory Standards Institute (CLSI). The multiple proportion dilution of the mentioned compounds was blended with log-phase bacterial cell suspension with final concentration of 1.5 × 10^6^ CFU/ml. The minimum inhibitory concentration (MIC) was determined by measuring OD_630_ with no visible turbidity after 16–20 h incubation at 37 °C (Yang et al. 2017).

### Kirby-Bauer (K-B) disk diffusion testing

As recommended by CLSI guidelines (Prevention 2018), the *E.coli* ATCC 25,922 and other clinical isolates were seeded onto a Mueller-Hinton (MH) plates surface employing sterile swabs and tamped prepared discs gently after adjusting to 0.5 McF of cell suspension. Due to the need to ensure consistent and quality amongst antibacterial agents and the lack of commercialized discs impregnated with tavaborole, 6 mm blank discs were purchased and infiltrated with equal amounts of antibiotics including tobramycin (TOB), amikacin (AMK), and polymyxin B. (PMB). DMSO was served as negative control. After incubating for 20–24 h at 37 °C in 5% CO_2_ surroundings, the area in which antibacterial agents completely inhibited bacterial growth was used for diameters measurement (Nakamura et al. 2014).

### Synergy screening of antibiotics library

A number of known and approved antimicrobial drugs consist of antibiotics library and the optimal combination with tavaborole against *E.coli* ATCC 25,922 was obtained through synergy screening. The overnight inoculum was diluted to around 1 × 10^6^ CFU/ml in MH. A 96-well round-bottom plate with 8 wells each concentration along the ordinate was pipetted with a double ratio dilution of tavaborole at 50 µl/well. Antibiotics were then added along the abscissa using the same dilution approach. Only MH media and DMSO were used in the background and sterility controls. Turbidity of each well was examined at 630 nm on the iMark™ microplate absorbance reader after 18-hour incubation at 37 °C (Ejim et al. 2011).

### FIC index determination

As previously noted, fractional inhibitory concentrations (FICs) were determined (Song et al. 2021). The MIC for each drug was established using a checkerboard experiment with eight distinct concentration gradients of tavaborole and antibiotics, and the lowest concentration causing no visible turbidity. The sum of the two FICs, namely, FIC index (FICI), was used to calculate the synergistic impact of each antibiotic. FICI is calculated using the following formula:

FIC index = FIC_A_ + FIC_B_ =$\frac{{MIC}_{AB}}{{MIC}_{A}}+ \frac{{MIC}_{BA}}{{MIC}_{B}}$ MIC_A_ is the MIC of tavaborole; MIC_AB_ is the MIC of tavatorole in the presence of antibiotics; MIC_B_ is the MIC of each antibiotic; MIC_BA_ is the MIC of antibiotics in the presence of tavaborole; FIC_A_ is the FIC of tavaborole; FIC_B_ is the FIC of each antibiotic. Based on the previous studies (Mahomoodally et al. 2018), the combination effects of tavaborole and a wide range of antibiotics were defined as below: FICI ≤ 0.5 was synergistic effect; 0.5 < FICI ≤ 1 was additive effect; 1 < FICI ≤ 4 was indifference; FICI > 4 was antagonistic effect.

### Time-dependent growth and killing curves of bacteria

Overnight cultures of *E.coli* isolations were cultivated in 5 ml of LB at 37 °C with shaking at 180 rpm, as directed by CLSI. The bacterial suspension was diluted 1/100 in fresh Mueller Hinton (MH) Broth medium after being adjusted to 0.5 McFarland. For a single drug time-dependent growth test, the cells were treated with tavaborole (1/4 × MIC to 8 × MIC). For growth and killing kinetic curves of combined medicines, cells were treated with tavaborole (4 µg/ml), aminoglycoside antibiotics (1/8 × and 1/4 × MIC) alone or in combination for 24 h at 37 °C 180 rpm. As a control, a 2% concentration of DMSO was used. 100 µl aliquots were removed to conduct turbidity measurement and ten-fold serially diluted suspensions were plated on blood agar plates for calculating the colony-forming units (CFUs) after incubation at 37 °C for 24 h at the time point of 0, 2, 4, 8, 12 and 24 h, respectively(Song et al. 2021).

### Drug resistance inducing assays

First, the MIC of drugs (tavaborole, TOB, AMK and RFP) was measured as described above. Then, after incubation at 37 °C for 18 h, the bacterial suspension at sub-MIC concentration was diluted 1:1000 into fresh MH media supplement with different concentration of drugs for testing next MIC passages, which was repeated for 30 days. The fold change of MIC relative to initial MIC was calculated (Liu et al. 2020). The drug resistance development of aminoglycoside antibiotics (TOB and AMK) and tavaborole combined was also conducted. In addition, the obtained resistant *E.coli* ATCC 25,922 were measured the cross-resistance to other kinds of antimicrobials using the same MIC assay. Each experiment was performed with replicates at least twice at intervals (Zhong et al. 2021).

### Biofilm formation evaluation

Biofilm formation of *E.coli* strains was evaluated by two methods. One of them was measured OD_630_ directly after removing supernatant and the other was crystal violet (CV) staining assay as described previously(Shi et al. 2018). Briefly, overnight cultures were adjusted to 0.5 McF in LB (total 200 µl) containing sub-MIC of tavaborole, aminoglycoside antibiotics (TOB and AMK) alone and their combination and grown in 96-well polystyrene microplates without shaking at incubator for 24 h, 48 and 72 h (6 duplicates per condition per experiment). Rinsed with 200 µl/well of 0.9% saline solution, immobilization with methyl alcohol for 15 min and the same volume of 0.25% CV per well was used to stain biofilm at room temperature for 15 min. Per well was washed three times with 0.9% NaCl solution and air-dried, subsequently, 95% ethanol was added to dissolve CV for 20 min. The absorbance was recorded at 570 nm using a microplate absorbance reader.

### Motility assays

Different agar concentration and nitrogen source was allowed for different motility assay. For swimming assay, medium was included 0.3% agar (wt/vol), 1% tryptone and 0.5% NaCl and plates were stab inoculated with mid-log-phase bacteria using sterile toothpicks. For swarming assays, medium consisted of 0.5% agar, 0.8% nutrient broth and 0.5% glucose and plates were spot inoculated with 2 µl of *E.coli* cultures. After inoculation, all plates were incubated for 14–16 h at 37  °C and imaged by UVP ChemStudio/PLUS (AnalytikJena, Germany) (Coleman et al. 2020; Tan et al. 2021).

### Hydrophobicity analysis

*E.coli* hydrophobicity was assessed by microbial adherence to hydrocarbon (MATH) test with minor modifications(Campbell et al. 2020). 5 ml of *E.coli* inoculum was grown in LB treated with tavaborole (4 µg/ml), TOB (0.5 µg/ml) alone and their combination for 24 h at 37 °C with shaking at 180 rpm. Then cultures were washed with 1 × PBS and diluted to an OD 600 of 0.3 (5 ml of total volume). 1 ml of suspension was removed for counting CFUs and 1 ml of n-hexadecane was added to the air–liquid interface to keep volume constant. Vortexed for 1 min and separated the phases for 15 min at room temperature, 1 ml of the lower aqueous layer was removed for counting CFUs. Results were interpreted as the percentage of cells excluded from aqueous layer and hydrophobicity level was evaluated by formula: $\frac{\text{A}0 - \text{A}1 }{\text{A}0}$×100%. A_0_ and A_1_ were the CFU counts before and after the addition of hexadecane. Hydrophobicity of *E.coli* strains were classified into three categories: highly hydrophobic (the values more than 70%); moderately hydrophobic (values ranging from 50 to 70%); and lowly hydrophobic (values less than 50%).

### Label free-based proteomic profiling

#### Sample preparation

*E.coli* ATCC 25,922 was overnight grown in 10 ml LB and passaged for 3 h with 1:50 dilution in fresh LB. Treated with tavaborole alone (80 µg/ml, labeled ‘A’) or tavaborole plus amikacin (80 µg/ml + 40 µg/ml, labeled ‘AK’) for 6 h, cells were washed in PBS and harvested by centrifugation at 4,000 g, 8 min. After liquid nitrogen flash freezing, cells were stored at −80 °C and transported to company for following experiments(Tian et al. 2020).

### Bacteria lysis and protein digestion

According to the filter-aided sample preparation (FASP) procedure(Wiśniewski et al. 2009), bacteria was lysed by SDT buffer (4% SDS, 100 mM Tris-HCl, 1 mM DTT, pH 7.6) and protein was digested by trypsin and quantified by BCA Protein Assay Kit (Bio-Rad, USA). The digest peptides of each sample were desalted on C18 Cartridges (Empore™ SPE Cartridges C18 (standard density), bed I.D. 7 mm, volume 3 ml, Sigma), enriched by vacuum centrifugation and reconstituted in 40 µl of 0.1% (v/v) formic acid.

#### LC-MS/MS

LC-MS/MS analysis was performed on a Q Exactive mass spectrometer (Thermo Scientific) coupled to Easy nLC (Proxeon Biosystems, now Thermo Fisher Scientific). Digested protein mixtures were loaded onto a reverse phase trap column (Thermo Scientific Acclaim PepMap100, 100 μm*2 cm, nanoViper C18) at a flow rate of 300 nl/min in buffer of 0.1% formic acid and 84% acetonitrile. The mass spectrometer was operated in positive ion mode. Survey of full-scan MS spectra (300–1800 m/z) were acquired with a resolution of R = 70,000 at m/z 200. Dynamic exclusion duration was 40.0 s. The raw data were combined and searched using the MaxQuant 1.5.3.17 software for identification and quantitation analysis as described previously (Pettersen et al. 2021).

### Hemolysis assay

Hemolysis assay was performed in accordance with previously reported protocols (Idowu et al. 2019). Purchased from the Hemo Pharmaceutical and Biological Co (Shanghai, China), human red blood cells (RBCs) were centrifuged and transferred into a 96-well plate. The final concentration of RBCs was up to 5% v/v and then treated with tavaborole at concentration of 4-128 µg/ml at 37 °C for 1 h. The hemolysis positive and negative controls were 0.1% TritonX-100 and 1% DMSO, respectively. The supernatant was taken to measure its absorbance at 570 nm. The concentration of 50% hemolysis of red blood cells (HC_50_) was as interpretation. The calculation formula was as followed:

Hemolysis (%) =$$\frac{{A}_{sample}-{A}_{Neg.}}{{A}_{Pos.}-{A}_{Neg.}}\times$$100%

### Cytotoxicity assay

To further evaluate the cytotoxicity of tavaborole, cell viability was evaluated by Cell Counting Kit-8 (DojinDo, Japan), including LO2 (human normal liver cell line), HepG2 (human liver cancer cell line), HMC3 (human microglial clone 3 cell line), U251 (human glioma cell line), HK-2 (epithelial cell line from human renal proximal convoluted tubule), 786-O cell line (human renal carcinoma cells). HK-2 cells were cultured in RPMI 1640 medium. LO2, HepG2, HMC3, U251 and 786-O cells were grown in Dulbecco’s modified Eagle’s medium (DMEM). All of cells were supplied with 10% fetal bovine serum (FBS) 1% L-glutamine and 1% penicillin/streptomycin solution in a 37 °C incubator containing 5% CO_2_. The optical density (OD) was read at 490 nm on a plate reader. More details were described previously(Hankittichai et al. 2020; Wang et al. 2020; Wu et al. 2020). The formula of counting cell viability as followed:

Viability (%) =$$\frac{{A}_{sample}-{A}_{blank}}{{A}_{0.1\%DMSO}-{A}_{blank}}\times$$100%

### Mice peritonitis infection model

This murine-related laboratory procedures were approved by the Ethics Committee of the Third Xiangya Hospital of Central South University (No.2021sydw0245). We mimicked a reported protocol with minor modification(Liu et al. 2020). Female ICR mice (n = 6) were intraperitoneally administrated with 5 × 10^5^ CFUs *E.coli* Y9633 (MDR) suspension. After 1 h post infection, mice were treated intraperitoneally a single dose of tobramycin (4 mg/kg), tavaborole (20 mg/kg) alone or tobramycin plus tavaborole (4 + 20 mg/kg) and were sacrificed by cervical dislocation after 24 h. The kidney, liver, lung and spleen were aseptically separated, homogenized, ten-fold serially diluted, and plated on Columbia blood plates to count bacterial quantity after incubated at 37  °C for 24 h (Luther et al. 2019).

### Histological examination

To assess the degree of tissue inflammation in peritonitis model and the systemic toxicity in vivo, the mice were sacrificed at the point of 24 h, and organs (heart, liver, spleen, lung, kidney) were scissored and fixed in 5 ml 4% neutral paraformaldehyde fixator solution (Servicebio, Wuhan, China) before executing hematoxylin and eosin (H&E) staining operation. Images were captured by microscope with random option locations (Zhao et al. 2019).

### Data analysis

Statistical analysis was performed by Prism 9.0 (GraphPad Software, San Diego, CA, United States). Except extra annotation, all the data was presented as mean ± standard deviation and statistical significance was analyzed by Student’s two-tailed t-test or one-way ANOVA. Proteomic profiling was analyzed by Fisher’s exact test. Differences between groups were determined significant when *p-*value is less than 0.05 (*p < 0.05, **p < 0.01, ***p < 0.001).

## Results

### Tavaborole and other Benzoxaborole Compounds have Antibacterial Properties

The drug library contained antibiotics as for positive control that known to kill bacteria. In Fig. 1a, “116” means the number of all drugs that inhibit bacterial growth and keep the liquid culture medium clear. “26” means the number of potential drugs among 116 drugs, excluding antibiotics and compounds that have already been studied. Finally, we focused on tavaborole after screening the drug library of 2049 chemicals. The antibacterial activity of tavaborole and other benzoxaborole compounds was assessed using the values of MIC listed in Table 1 and the chemical structural formula of these benzoxaborole compounds were represented in Fig. 1b. The fluorine atom of tavaborole was coupled to the benzene ring, whereas the chlorine atom was bonded to the benzene ring of AN-2718. As shown in the Table 1, tavaborole and AN-2718 were equally active against *E. coli* and *S. aureus*, with MICs ranging from 8 to 16 µg/ml and 16–64 µg/ml, respectively. Another benzoxaborole molecule, Crisaborole, had a wide range of antibacterial activity against G + bacteria (MIC = 4–64 µg/ml), but only a modest antibacterial activity against G- bacteria (MIC = 64 µg/ml). In conclusion, benzoxaborole structure is the critical part in compounds that had antimicrobial activity against gram-negative and gram-positive microorganisms. But different side chains would alter the efficiency of antimicrobial active substances. Our research target for further investigation was *E. coli*.

As per the growth curve, tavaborole inhibited cell growth for 8 h at 4 µg/ml, (Fig. 1c). Antibacterial activity of compounds was studied in susceptibility tests with 3 *E. coli* strains using the disc diffusion method. The inhibition zone diameters of tavaborole disc spanned from 22 to 24 mm, suggesting tavaborole had no strain-specific activity. Moreover, the inhibition area of tavaborole was larger than that of other conventional antibiotics (such as TOB, AMK, and PMB) in *E. coli* Y9395 and Y9633 (Fig. 1d-i). The margin of the inhibition area of tavaborole was indistinct, but the PMB border was obvious, indicating tavaborole was a growth inhibitor with no bactericidal effect (Fig. 1d-f). In summary, tavaborole was found to be a bacteriostatic benzoxaborole molecule that was effective against *E. coli*.

**Table 1:** MIC (µg/ml) of tavaborole, AN-2718 and Crisaborole against Gram-Positive and Gram-Negative Bacteria

Strain	Tavaborole		AN-2718		Crisaborole	
*S.aureus*						
Newman ^a^	16		64		32	
ATCC 43,300 ^a^	32		32		4	
LZB1 ^a^	32		32		32	
USA 300 ^a^	32		32		8	
RJ-2 ^a^	64		64		64	
SAJ-1 ^a^	16		16		16	
*S.epidermidis*						
RP62A	16		16		32	
*E.coli*						
ATCC 25,922	8		8		> 64	
Y9633^b^	16		16		> 64	
Y0064^b^	16		16		> 64	
Y9592^b^	8		8		> 64	
Y9395^b^	8		8		> 64	
* K.pneumoniae*						
ATCC 700,603	32		32		> 64	
WANG^b^	32		32		> 64	
* A.baumannii*						
ATCC 1195	16		> 64		> 64	
*P.aeruginosa*						
PAO1	32		32		> 64	

^a^Represents Methicillin-resistant Staphylococcus aureus

^b^Represents multidrug resistant strains

### Tavaborole increases the effectiveness of AGAs in MDR ***E. coli***.

**Fig. 2:**
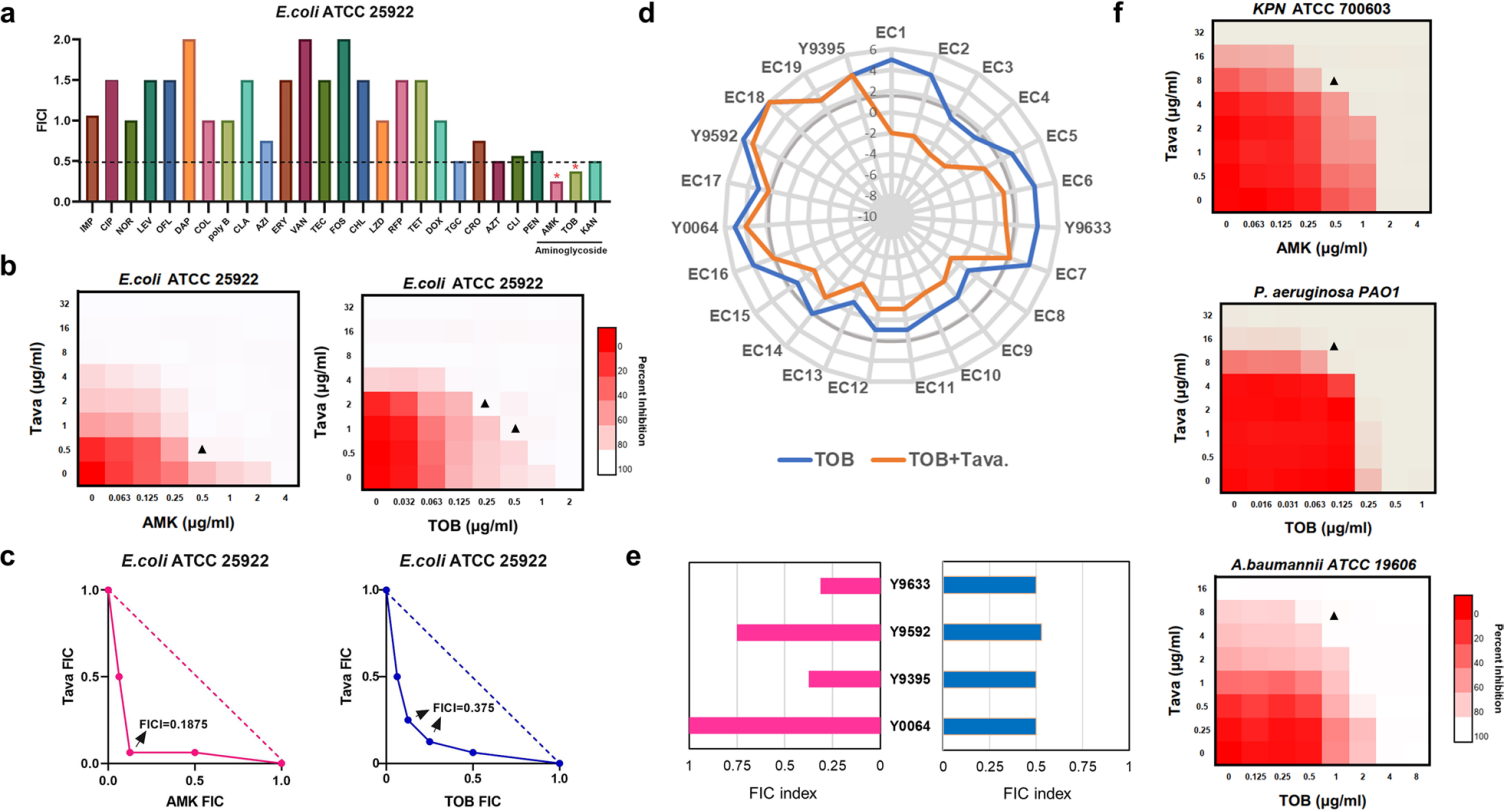
Tavaborole boosts the efficacy of AGAs against MDR *E. coli*. **a** Antibiotics library combination screening. Synergistic antibiotics are indicated by a red star. **b** Microdilution tests between Tava and AMK or TOB on a checkerboard. Dark-red regions represent higher bacteria loading and lower growth-inhibition capacity. The X- and Y-axes were both set to log2 scale. **c** FIC plot for AMK or TOB in conjunction with tavaborole. The additive impact is represented by the dotted line. The point utilized to calculate FICI is indicated by black arrows. **d** Changes in MICs against clinical MDR *E. coli* infections between TOB therapy (blue line) and TOB plus 1/2 ×MIC Tava treatment (orange line). The log2-transformed is shown on the radial axis. Breakpoint (4 g/ml) is indicated by the grey thick line. **e** The FICI are calculated in partial clinical isolates. **f** Checkerboard dilution method of tavaborole combined with AMK against *K. pneumoniae* ATCC 700,603, and with TOB against *P. aeruginosa* PAO1 and *A. baumannii* ATCC 19,606. Three biological replicates are used in each experiment. Synergy is defined as an FIC index of ≤ 0.5

We analyzed the FICI of tavaborole and several antibiotics using the Checkerboard assay and the results showed synergistic interaction with AGAs (Fig. 2a). With an 8-fold decrease in MIC values from 4 µg/ml to 0.5 µg/ml and 2 µg/ml to 0.25 µg/ml, respectively, tavaborole had the highest synergistic effectiveness with AMK (FICI = 0.1875) and TOB (FICI = 0.375) (Fig. 2b-c). We then explored the MIC changes of TOB after tavaborole treatment to see if the synergy was a generalized phenomenon in *E. coli* resistant infections. As previously stated, MICs decreased and converged near the breakpoint. While the MICs of three pathogens remained unaltered, representing the synergistic effect was strain-specific (Fig. 2d). In these strains, synergistic and additive impact between tavaborole and TOB or AMK was identified (Fig. 2e and Additional file 1: Figure S1). Additionally, tavaborole influenced the effectiveness of AMK or TOB against other G^−^ bacteria (Fig. 2f and Additional file 1: Figure S2). Overall, tavaborole with strain-specific synergistic properties was discovered to be a possible adjuvant to AGAs, particularly TOB and AMK.

### Tavaborole enhances AGAs efficacy and pharmacodynamic parameters changes

**Fig. 3:**
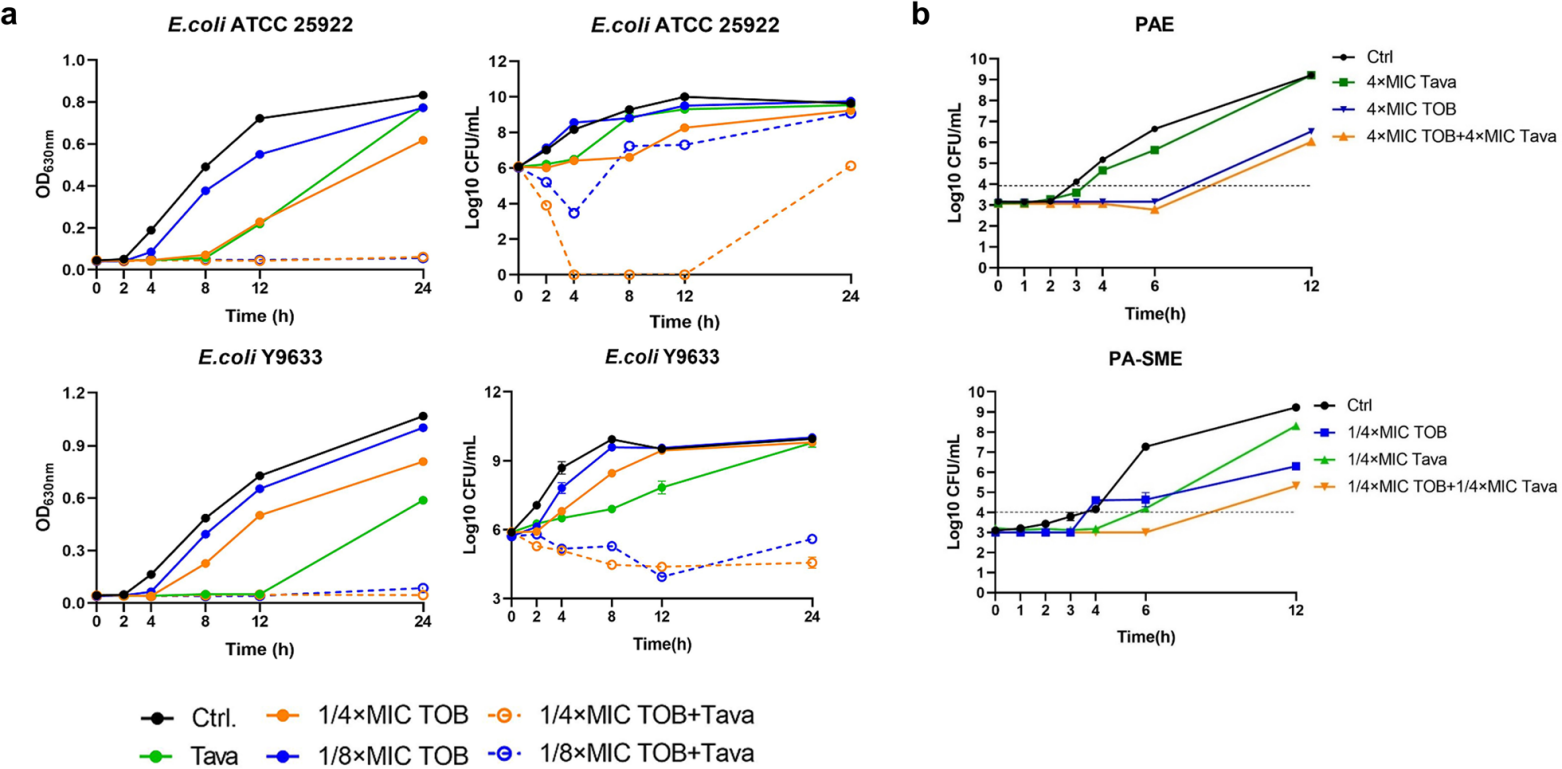
The growth, killing and kinetic curves of tavaborole coupled with AGAs. **a** Growth and killing curves of *E. col*i ATCC 25,922 and Y9633 treated with DMSO (Ctrl), tavaborole (Tava, 4 µg/ml), or tobramycin (TOB, 0.5 or 0.25 µg/ml) alone or in combination (Tava + TOB, 4 µg/ml + 0.5 µg/ml or 0.25 µg/ml). At intervals, the CFU/m of bacterial was determined. **b** PAE and PA-SME of Tava plus TOB (16 µg/ml + 4 µg/ml) in comparison with untreated control cultures and 1–4 × MIC of Tava or TOB alone against *E. coli* ATCC 25,922. All the trials were carried out three times

Although the results of FICI suggested that tavaborole potentiated the effectiveness of TOB and AMK, synergistic bactericidal curve may strengthen these findings. Therefore, time-dependent growth and killing experiments against *E.coli* ATCC 25,922 and *E. coli* Y9633 was conducted by treated with tavaborole, TOB (or AMK), both thereof. We noticed that single pharmacotherapy had weak inhibitory effects, but adding tavaborole (4 µg/ml) into the concentration with sub-MIC of TOB (or AMK) apparently strengthened bactericidal activity (Fig. 3a and Additional file 1: Figure S3).

On the basis of recent study, the post-antibiotic effect (PAE) and post-antibiotic sub-MIC effect (PA-SME) were the most common pharmacodynamic parameters in vitro (Oh et al. 2019). The synergy data and kill-curve assay were used to determine the corresponding parameters of PAE and PA-SME (Gaibani et al. 2014). About PAE, Fig. 3b showed the average amount of time in 32 µg/ml tavaborole and 8 µg/ml TOB (both corresponding to fourfold MIC) was nearly 0.5 and 4.6 h, respectively, however, the duration of bacterial inhibition in combination treatment revealed no discernable extension. About PA-SME, the duration of bacterial inhibition in combination treatment was longer than that of time at a subinhibitory concentration (1/4 × MIC) of TOB. These findings demonstrated that tavaborole may have a beneficial impact on TOB dosage regimen and we were planning a follow-up study to prove that.

### Synergistic treatment has lower propensity for resistance development

**Fig. 4:**
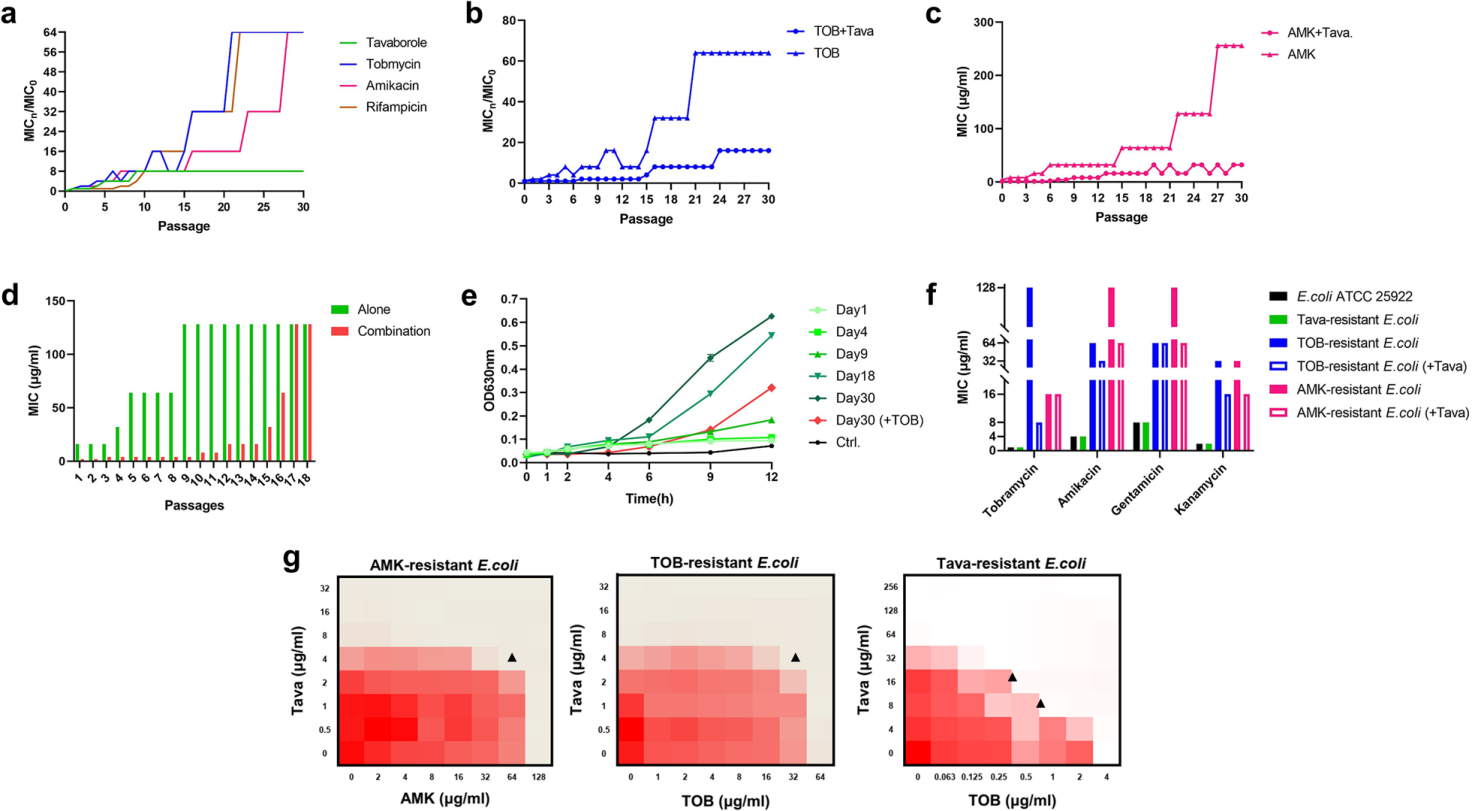
The resistance progression of tavaborole coupled with AGAs. **a** Resistance development of *E. coli* ATCC 25,922 in the presence of sub-MIC concentration of Tava, TOB, AMK, and RFP. TOB (**b**) and AMK (**c**) resistance was acquired with or without the presence of tava (2 µg/ml). **d** MIC changes of Tava alone and in the combination with TOB at days 1–18, respectively. **e** Growth curves of one of the evolving *E. coli* cultures collected at day 1, 4, 9, 18, 30 and regrown simultaneously in the presence of Tava (16 ug/ml). **f** MICs comparison graph of tobramycin, amikacin, gentamicin, and kanamycin against parental strains and induced drug-resistant bacterial strains. **g** Checkerboard graph of Tava combined with TOB or AMK to kill induced drug-resistant bacterial strains. Black arrows indicate the point used to calculate FICI. All the trials were carried out three times

To investigate whether tavaborole likely to induce *E. coli* ATCC 25,922 mutations, the rate of inducing resistance was measured with traditional antibiotics for contrast. As shown in Fig. 4a, there was little changed in MIC of tavaborole during 30 passages while a 64-fold increasement of MIC was observed in TOB, AMK and RFP treatment indicating tavaborole had the advantage of lower resistance that the bacteria are induced to produce. In comparison of monotherapy, the resistant rate of TOB and AMK was dropped with 8-fold decrease when adding 1/4 × MIC tavaborole (Fig. 4b-c). Besides, we found TOB had influence on tavaborole resistance and it took 8 days longer to reach 128 µg/ml than tavaborole monotherapy (Fig. 4d). The evolving *E. coli* cultures that were collected on specific days that tavaborole treated and the growth curves were determined in the present of 16 µg/ml tavaborole (Fig. 4e). The initial (day 1) and parent (control) strains were growth arrested while the evolving strains grew at an ascendingly rapid rate under the same conditions, denoting resistance had been acquired successfully.

Following that, we measured the MICs of resistance inducing strains to identify whether the cross-resistance phenomenon was existed. In Fig. 4f, cross-resistance between AGAs was observed in TOB- and AMK-resistant *E. coli*, however evolving strains with combined treatment showed significant decrease of MICs. Similarly, regardless of single or combined tavaborole-resistance *E. coli*, the MIC of ampicillin increased, whereas the MICs of high molecular weight antibiotics (such RFP, ERY, and CLA) decreased (Additional file 1: Figure S4). The synergistic impact was disappeared against TOB- and AMK-resistant *E. coli* collected at day 30, as indicated in Fig. 4g and Additional file 1: Figure S5, but there was no change in tava-resistant *E. coli* collected.

### Effect of Tavaborole on the Physicochemical Properties and Biofilm formation capability

**Fig. 5:**
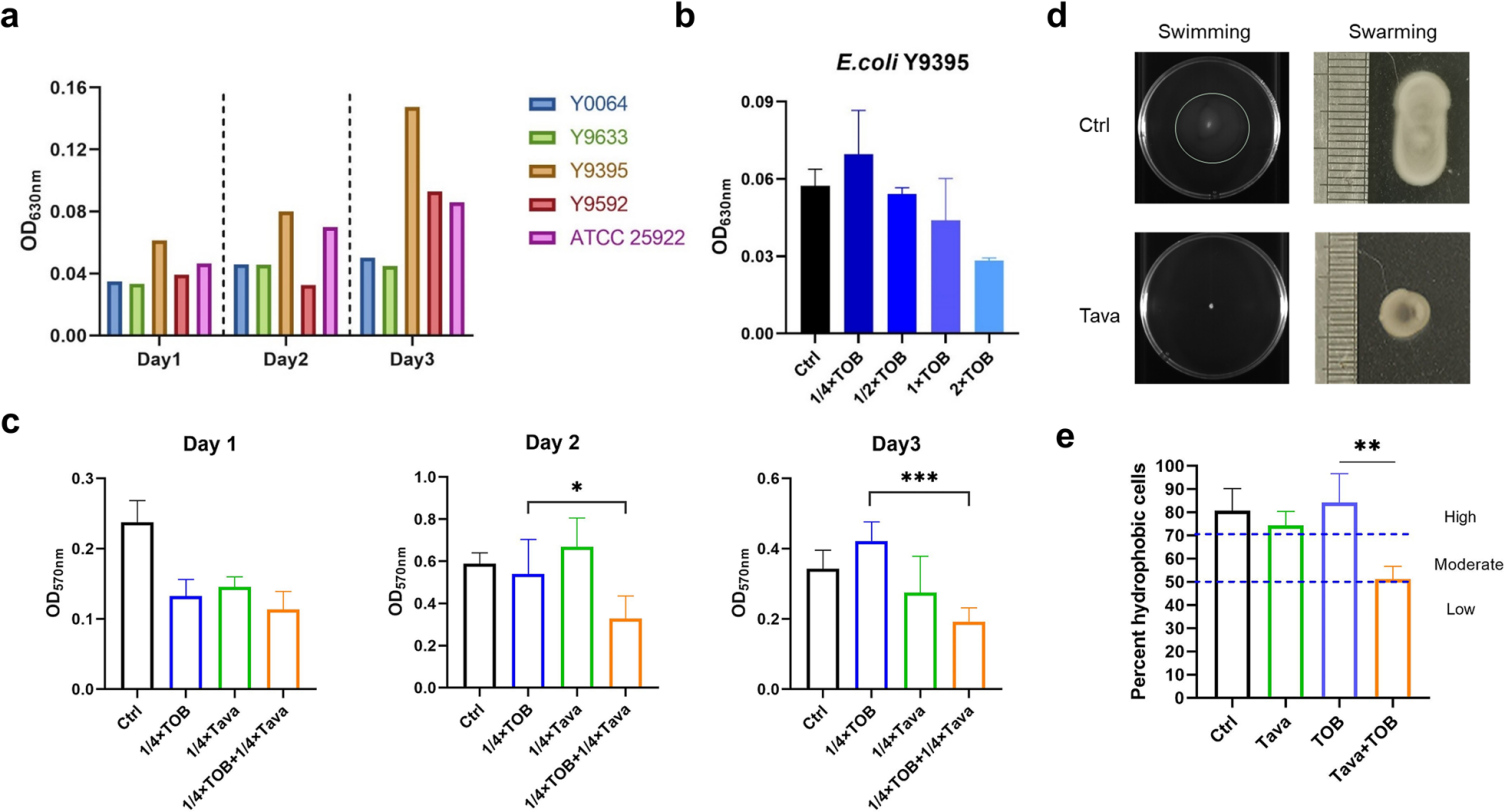
Tavaborole inhibits biofilm formation and motility. **a** The ability of *E. coli* to generate biofilms was assessed. **b** Biofilm development of *E. coli* Y9395 after treatment with 0.25-2 µg/ml TOB. **c** Effects of Tava (1/4 × Tava, 2 µg/ml), TOB (1/4 × TOB, 0.5 µg/ml), and the combination (1/4 × TOB + 1/4 × Tava, 0.5 µg/ml + 2 µg/ml) on biofilm inhibition at days 1–3. **d** Assay for swimming and swarming. *E. coli* ATCC 25,922 was grown to mid-log phase and inoculated 0.3% or 0.5% agar plates with DMSO (Ctrl) or Tava (4 µg/ml), respectively. **e** Effect of Tava or its combination with TOB on the hydrophobicity of *E. coli*. Bacterial hydrophobicity is classified as low (≤ 50), moderate (50–70%) or high (≥ 70%). DMSO was as a negative control. Error bars indicate standard deviation. All experiments are in triplicate. *p < 0.05; **p < 0.01; ***p < 0.001

As previously documented, Biofilm-encased bacteria likely were 10–100 times more than planktonic counterparts to be resistant to conventional antibiotics, inducing recalcitrance and recurrence of biofilm-associated uropathogenic infections (Beebout et al. 2019; de Breij et al. 2018). Besides, it’s been reported that flagella were associated with virulence and played an essential role in colonization, adherence and distribution (Jia et al. 2021; Wu et al. 2022). When used *E. coli* Y9395 for the biofilm associated experiments because of its higher biofilm formation capability (Fig. 5a). The results matched past studies that TOB stimulated biofilm formation at 0.25 µg/ml (1/4 × MIC) (Hoffman et al. 2005). Nonetheless, when synergized with tavaborole, TOB was able to inhibit biofilm development and the inhibitory effect was stronger by time (Fig. 5b-c).

In order to identify the inhibitory activity of motility, the swimming and swarming motility assays were performed. We observed bacteria was substantially restricted movement by 4 µg/ml tavaborole (Fig. 5d). Moreover, hydrophobicity influenced bacterial adhesion, proliferation and biofilm formation in variety of ways (Danchik and Casadevall 2020; Wu et al. 2022). When TOB was combined with tavaborole, the percentage of hydrophobic planktonic *E. coli* was reduced from 84 to 49%, compared to TOB monotherapy (Fig. 5e). Taken together, tavaborole had the ability to inhibited biofilm formation and decreased motility, suggesting tavaborole avoided further deterioration of disease and aided clinical therapy to an extent.

### Tavaborole in Combination Pharmacotherapy: Multifaced Mechanisms of Action

**Fig. 6:**
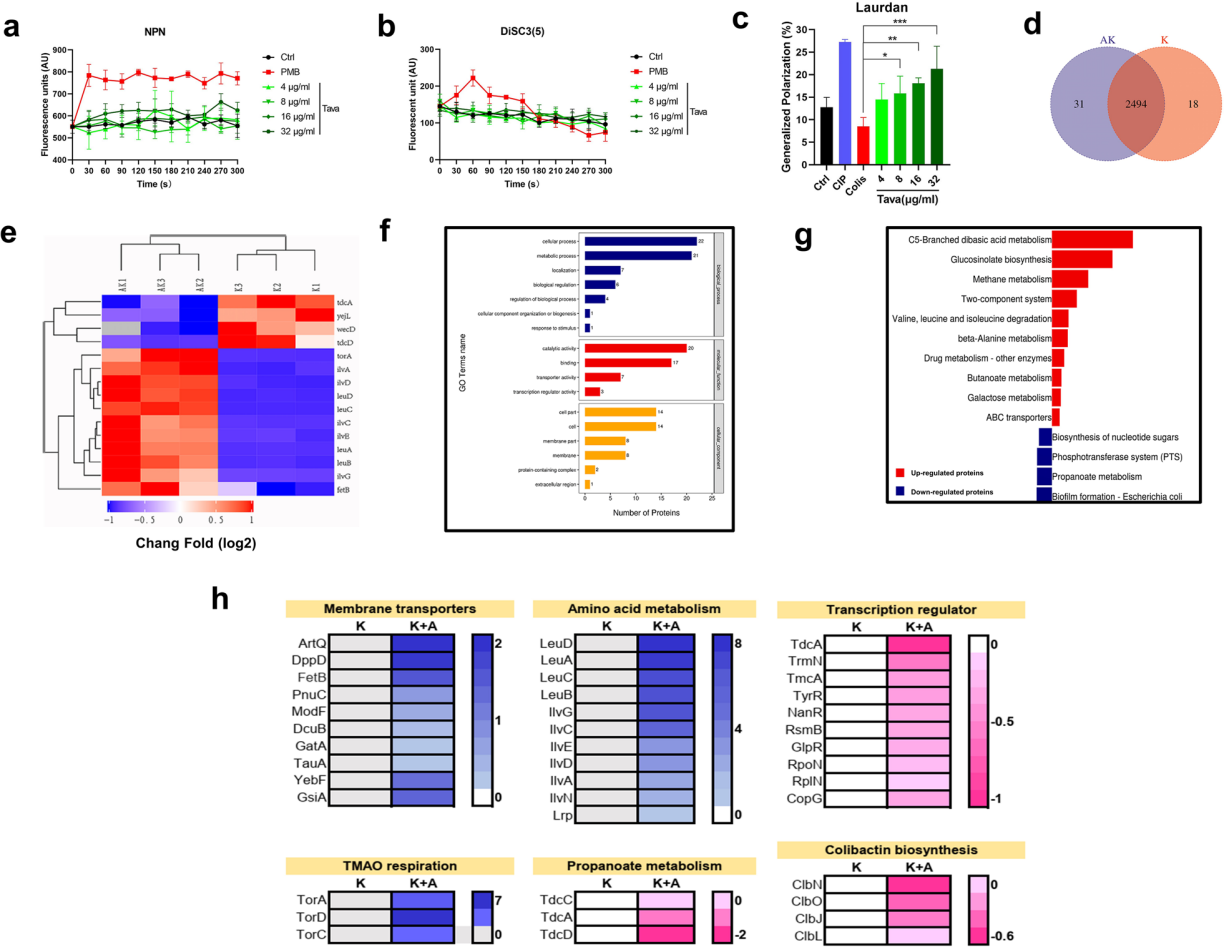
Multifaced mechanisms of synergistic action. By measuring the fluorescence intensity of **a** NPN and **b** DiSC_3_(5), tavaborole shows no disruption and dissipation to the outer membrane and membrane potential in *E. coli ATCC* 25,922. **c** Membrane fluidity was measured using the generalized polarization (GP) index after treatment with a serial concentration of tavaborole. Ciprofloxacin (CIP) and polymyxin B (PMB)were served as different positive controls. **d** Detection of intracellular ATP levels following a 1-hour treatment with 4–32 µg/ml tavaborole. **e** Heatmap of differential expression analysis demonstrating changes in primary protein regulation in *E. coli* ATCC 25,922 treated with amikacin (K) or amikacin-tavaborole (AK). There were two replicates in each group. Fisher’s exact test was used for comparison between groups. With a cutoff value of | log2 (fold change) | and *P* value of 0.05, up- or down-regulated expression is defined. **f** Annotation of differentially expressed proteins (DEPs) using Gene Ontology (GO). **g** Enrichment analysis of upregulated and downregulated DEPs using the Kyoto Encyclopedia of Genes and Genomes (KEGG). (h) DEGs about membrane transporter, TMAO respiration, amino acid metabolism, propanoate metabolism, transcription regulator, and colibactin biosynthesis are shown. K stands for amikacin, while K + A stands for amikacin with tavaborole (AN2690). *p < 0.05; **p < 0.01; ***p < 0.001

Based on earlier findings, we first investigated the effect of tavaborole on permeability of outer membrane (OM) using NPN and fluctuation in membrane potential using DiSC_3_(5). NPN, a hydrophobic fluorescent probe, interacted with hydrophobic regions of the phospholipid bilayer and produced fluorescence (Ning et al. 2017). DiSC_3_(5) is a membrane potential sensitive dye that has been linked to the proton motive force (PMF) (Hamamoto et al. 2015). Nevertheless, when exposed to tavaborole, we detected no significant increase in fluorescence (Fig. 6a-b).

Next, in order to figure out whether tavaborole disturbed membrane in other ways, we utilized Laurdan, a polarity-sensitive fluorescent probe for providing information on membrane heterogeneity (Bessa et al. 2018; Gunther et al. 2021). Ciprofloxacin is DNA topoisomerases inhibitor that interfered DNA replication and transcription and increased bacterial membrane heterogeneity. Whereas polymyxin B led to a loss of membrane integrity and decrease membrane fluidity. Surprisingly, we discovered that tavaborole caused a considerable rise in generalized polarization (GP) value, which indicated tavaborole impacted solidification of *E. coli* membranes (Fig. 6c). Following that, we used chemiluminescence to evaluate the intracellular levels of adenosine triphosphate (ATP) and found fluorescence increasement in a concentration-independent manner (Fig. 6d). Based on previous study, tavaborole likely to be a noncompetitive inhibitor with respect to ATP. That is the reason why the ATP content was not increasing proportionally with increasing tavaborole concentrations. Given the above results, we speculated that tavaborole could enhance membrane protein synthesis and active transportation.

We undertook proteomics analysis following exposures to amikacin (K) or amikacin-tavaborole (AK) combination for 6 h to acquire better understanding of the molecular mechanisms and the induced protein expression changes at the translation level. As our expectation, two groups revealed distinct proteinic expression patterns, confirming our hypothesis that tavaborole impacts protein expression contributing to mis-regulation of essential physiological process and cell growth suppression (Fig. 6e and Additional file 1: Figure S6). These differentially expressed proteins (DEPs) were split into three categories based on their GO annotations: biological processes, molecular function, and cellular component, among which membrane component, transport activity, and transcription regulator activity showed significant difference (Fig. 6f and Additional file 1: Figure S7a–c). In addition, KEGG enrichment analysis revealed that these DEPs were strongly enriched in amino acid biosynthesis (particularly leucine, isoleucine, and valine) and membrane transporters, whereas downregulated DEPs were associated with propanoate metabolism, biofilm formation, and other processes (Fig. 6g and Additional file 1: Figure S8a, b). Especially, the expression of TorCAD system increased by more than 100-fold and transport-related proteins were also upregulated after tavaborole treated. The transcription related regulators were downregulated (Fig. 6h).

### Tavaborole Combined with Tobramycin attenuates bacterial loading in mice Peritonitis Model

**Fig. 7:**
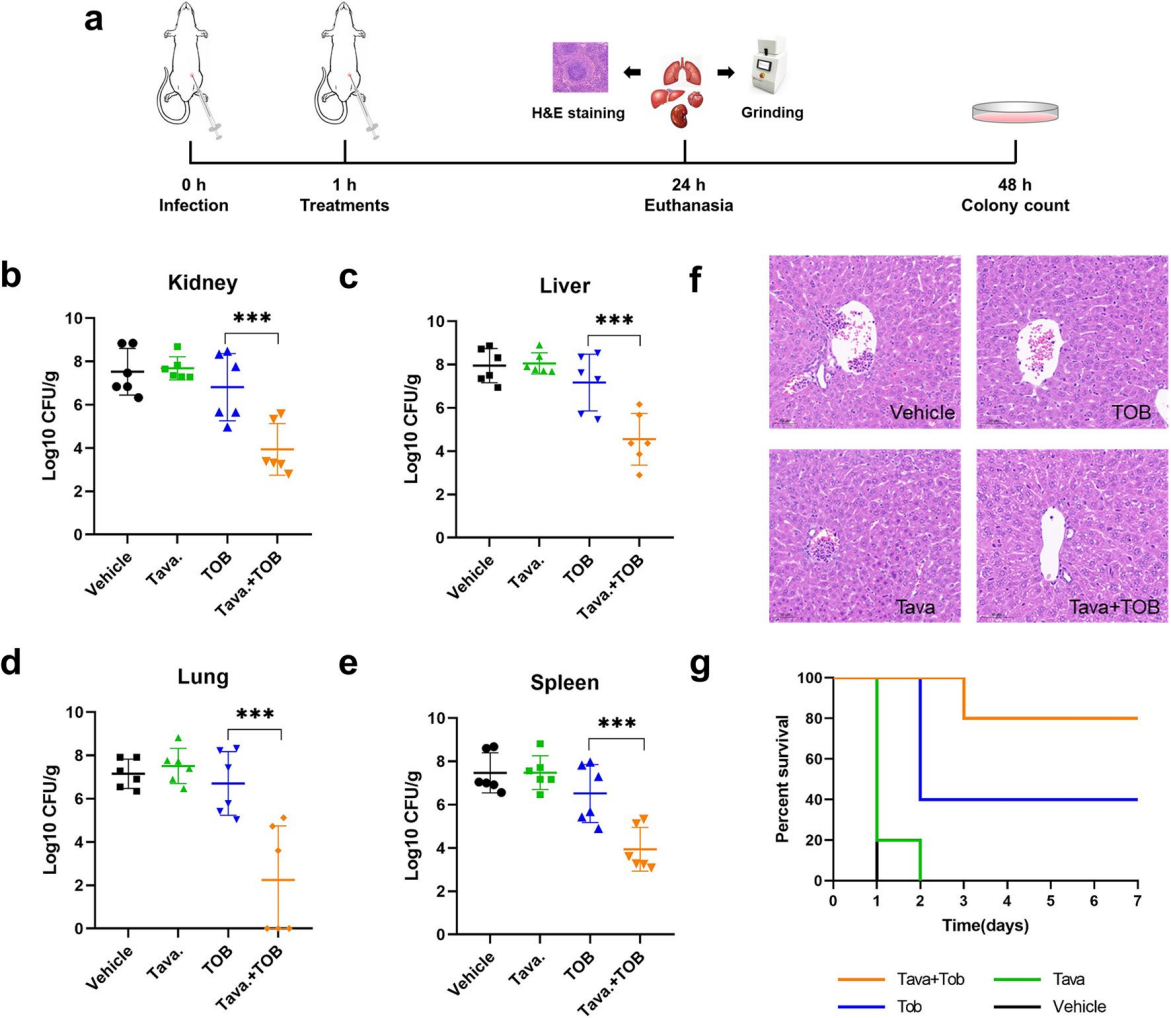
The synergistic activity in mice peritonitis model. **A** Flowchart for the formation of peritonitis model infected MDR *E. coli* Y9633 and the treatment regimen was followed. Agar plate counting of *E. coli* in mouse **B** kidney, **C** liver, **D** lung, and **E** spleen homogenate. ***p < 0.001. The graphs were the result of averaged three separate trials. **F** H&E staining pathological slice of mouse liver (the plotting scale is 50 μm). Inflammatory cell infiltration and bacterial burden can be effectively reduced in the combination group. Vehicle represented DMSO injection. The group that received 20 mg/kg of tavaborole was referred to as Tava. TOB and Tava + TOB served as 4 mg/kg TOB-treated group and 20 mg/kg Tava + 4 mg/kg TOB, respectively. **G** For 7 days, the survival rates of peritonitis mice treated with various dosing regimens had been observed

The antimicrobial effectiveness in vivo was assessed using an acute peritonitis model infected with *E. coli* Y9633 according to the protocol given in Fig. 7a. The acute peritonitis model was successfully created by counting the number of bacterial cells in the kidney, liver, lung, and spleen, and that the number of bacterial cells in the combination-treated group was significantly lower than the others, implying synergetic antimicrobial effects were also available and terrific in vivo (Fig. 7b-e). The liver pathological tissue slices were produced as indicated in Fig. 7f. Except for the combination treatment, inflammatory cell infiltration and aberrant hepatic cell arrangement were noticeable in other groups. Moreover, after 7 days, the combined group had an 80% survival rate, but the vehicle and tavaborole monotherapy groups had no survival on day 1 and day 2, respectively. Compared with TOB single treatment, the combined group significantly improved the animal survival rate, lowering death from 60 to 20%. (Fig. 7g).

### Systematic toxicity Assessment of Tavaborole

**Fig. 8:**
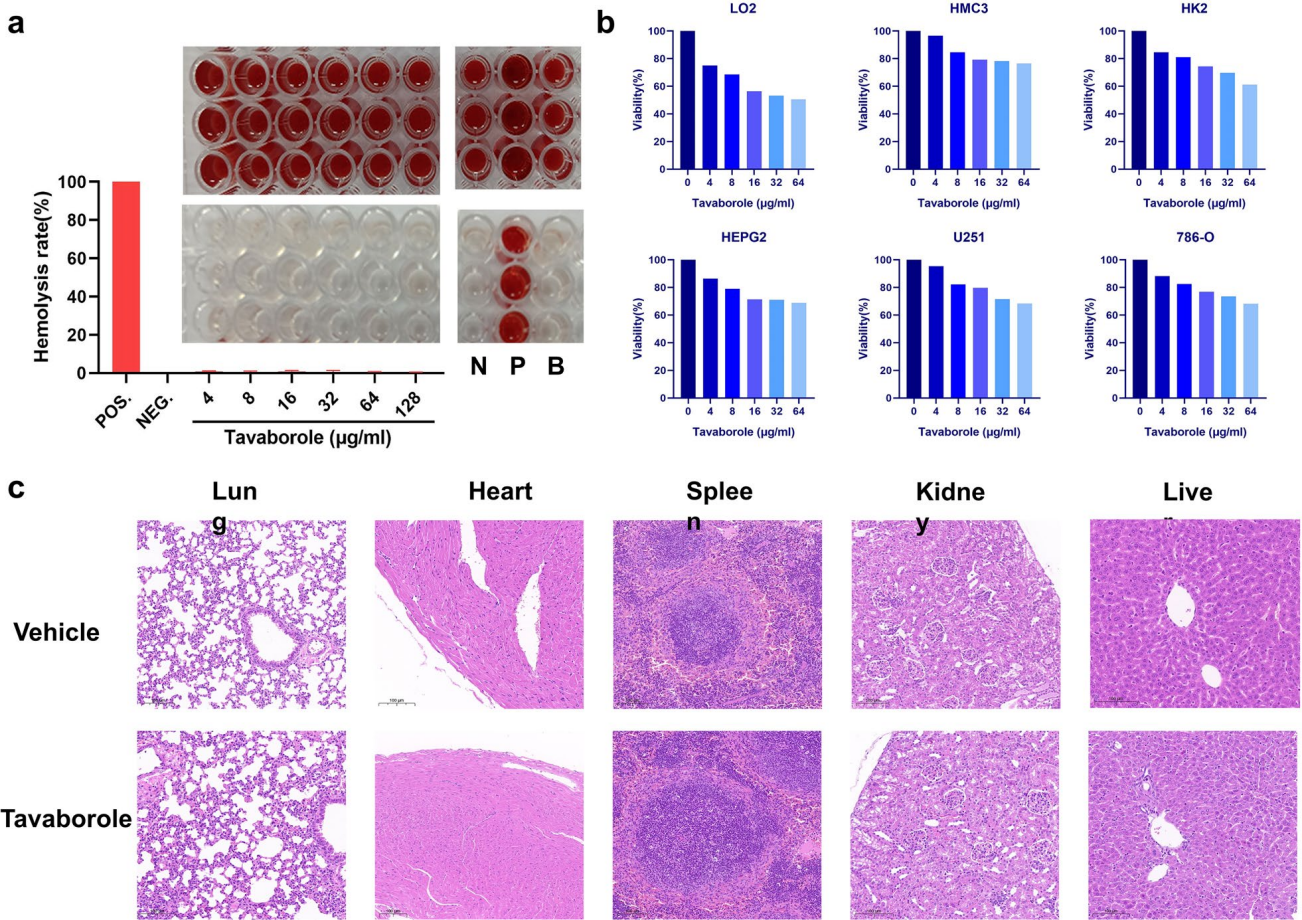
Toxicological examination of tavaborole. **a** Human red blood cells hemolysis rate of tavaborole. 0.1% Triton X-100 was served as positive control (“P”). 0.2% DMSO and PBS served as negative control (“N”) and blank control (“B”), respectively. **b** CCK-8 tests were used to determine the cytotoxicity of tavaborole to HMC3, U251, HK2, 786-O, HepG2, and LO2 cell lines. **c** H&E staining was used to compare pathological alterations in organ structure (lung, heart, spleen, kidney, liver) between the vehicle and tavaborole groups. Scale bars, 100 μm

The efficacy of tavaborole in vitro and its synergistic activity in vivo encouraged us to further investigated associated toxicity that would be presented by three methods. First one is hemolysis assay, tavaborole had no effect on red blood cells, and no hemolysis was observed even at 128 µg/ml (corresponding 16-fold MIC) (Fig. 8a). Second is cytotoxicity assay, tavaborole had 50% inhibitory concentration (IC_50_) values greater than 64 µg/ml in the HMC3, U251, HK2, 786-O, HepG2 cell line. The cancer cells were metabolically active and sensitive to external stimulation. These results suggested tavaborole had extremely low neurotoxicity and nephrotoxicity. The IC_50_ of tavaborole in the LO2 cell line was around 64 µg/ml (8 × MIC), but the concentration was higher than the dosage used and still within the acceptable level (Fig. 8b).

Finally, we investigated acute toxicity in mouse model. The mice were given 20 mg/kg tavaborole for 24 h before being euthanized for histopathology studies. Histological microstructures of the lung, heart, spleen, kidney, and liver in the medicated group were similar to unmedicated group and revealed no significant structural abnormalities (Fig. 8c). Additionally, based on the findings of carcinogenicity investigations and genotoxicity testing, no carcinogenesis, mutagenesis, or impairment of fertility were found in tavaborole (Ciaravino et al. 2014; Ciaravino et al. 2013; RxList 2018). To summarize, tavaborole had negligible systemic toxicity and was considered to be of great potential in clinical applications.

## Discussion

With the rise of aminoglycoside-resistant G- uropathogens, a new antibacterial agent is needed to overcome the globalization conundrum (Foxman 2010; Wagenlehner et al. 2019). We identified a benzoxaborole molecule tavaborole with antibacterial and synergistic efficacy simultaneously using a HTS assay, which provided a convenient and efficient method to repurpose medicine often used in other diseases treatment (Poole et al. 2018). There are several reasons why we choose 0.5 McF without dilution for HTS assay. On the one hand, high concentration of bacteria is beneficial to minimize the error existing among wells and plates and reduce the growth difference. On the other hand, we can preliminarily determine whether the compound is bactericidal or bacteriostatic. If it is bactericidal, the corresponding well will keep clear. If it is bacteriostatic, the turbidity is much lower than that of negative control, although there is still bacterial growth. According to our research data, the highly concentration-dependent bactericidal efficacy of combination supplied a substantial base of therapeutic scheme, which was assessed in diverse G- bacteria and 23 MDR *E.coli* strains.

As we all know, antibacterial drug resistance is a sophisticated and multifaceted problem, and the speed with which new drugs are developed is being massively surpassed by resistant evolutionary pressures (Idowu et al. 2019). In our research, we discovered tavaborole delays resistance evolution of AGAs and mitigates cross-resistance phenomenon. Likewise, AGAs slow tavaborole resistant rate. Then there is the link between experimentally acquired resistance and adaptive therapy (Igler et al. 2021). Adaptive therapy aimed to deal with the development of medication resistance by regulating disease rather than eliminating microorganisms (Hansen et al. 2020). In recent studies, tavaborole was proposed for using adaptive therapy with norvaline, which was harmful to human health, and we first identified AGAs had the potential to substitute norvaline in clinical application (Melnikov et al. 2020).

Based on previous studies, bactericidal mechanism of AGAs was mistranslation by disturbing ribosome function (Bruni and Kralj 2020) and internalization of AGA was significantly reliant on ∆ψ and energy (Leviton et al. 1995; Ramirez and Tolmasky 2010). This study, we have proven tavaborole possessed multifaced mechanisms among which accelerates AGAs uptake and promotes protein mistranslation. Tavaborole boosts energy production and increased TorCAD system expression, which provided 6 H^+^ to the PMF per oxidized NADH molecule, equating to the generation of 5 × 10^22^ ATP molecules (Denby et al. 2015; Qin et al. 2021). Tavaborole indirectly influenced PMF via regulating associated protein expression that is to explain why DiSC_3_(5) remained unchanged in short period. Furthermore, we discovered tavaborole downregulates transcription related proteins and promotes leucine production in feedback regulation. These molecular processes work together to enhance mistranslation of AGA.

The MIC of tavaborole for *E.coli* Y9633 is lower than that of tobramycin in Additional file 1: Figure S1, but the used dose of tavaborole is higher (20 mg/Kg). To be honest, it was as much as our confusion that tavaborole with low MIC used higher dose in vivo and we attempted to explain it. Because of the staggering complexity of the in vivo environment, the MIC value in vitro was not entirely reflective of internal situation. We should further explore the in-vivo model of pharmacokinetic/pharmacodynamic (PK/PD) to maximize the use of in vitro time-kill and in vivo animal experiments data. Besides, pharmaceutical carriers, biological effect of itself, approach of administration and so on should be considered.

Tavaborole demonstrated no hemolysis and modest cytotoxicity in vitro, and no notable alterations in organizational structure in vivo. However, we must pay special attention to individuals with liver disease, and practical solutions to this problem include supplementation of liver protecting agents or molecular modification of tavaborole, which is our future research direction. Meanwhile, in an *E.coli*-infectious peritonitis model, tavaborole coupled with tobramycin showed outstanding antibacterial activity when compared to tobramycin alone.

In summary, our aim of this study is to develop a workflow that can be used for high-throughput screening for repurposing drugs. The current study is the first research work on tavaborole synergy with AGAs and to comprehensively explore the multifaced mechanisms of tavaborole in combination pharmacotherapy. Tavaborole is a potential drug candidate that has bacteriostatic action, inhibits resistance evolution of AGAs and has good safety profile. These findings imply that tavaborole could be a promising aminoglycoside adjuvant in the fight against therapeutically relevant pathogenic microorganisms. Meanwhile, the discovery of tavaborole motivates us to pursue molecular designs and the discovery of other benzoxaborole structure molecules with collaborative mechanisms as prospective antibiotic adjuvants. The current study is the first research work on tavaborole synergy with AGAs and to comprehensively explore the multifaced mechanisms of tavaborole in combination pharmacotherapy. Tavaborole has bacteriostatic action, inhibits resistance evolution of AGAs and has good safety profile.

## Supplementary Information

**Additional file 1: Figure S1.** Chequerboard microdilution assays of AMK or TOB and tavaborole against E. coli Y0064, Y9395, Y9592, and Y9633 (all of them were XDR strains). Higher bacteria loading and lower growth-inhibition ability are represented by dark-red regions. X- and Y-axes were as log2 scale. The experiment was conducted with three biological replicates.** Figure S2.** Checkerboard dilution method of tavaborole combined with TOB against K. pneumoniae ATCC 700603, and with AMK against P. aeruginosa PAO1 and A. baumannii ATCC 19606. The experiment was conducted with three biological replicates. Synergy is defined as an FIC index of ≤ 0.5.** Figure S3.** Time-dependent growth or killing curves of E. coli ATCC 25922 treated with DMSO (Ctrl), tavaborole (Tava, 4 μg/ml) or sub-MIC of amikacin (AMK, 2 or 1 or 0.5 μg/ml) alone or in combination (Tava +AMK, 4 μg/ml + 2 μg/ml or 4 μg/ml + 1 μg/ml or 4 μg/ml + 0.5 μg/ml). The bacterial CFU/mL at specific time points during 24 h were determined. The experiment was performed with three biological replicates. **Figure S4.** Sensitizing effect is calculated by fold reduction of antibiotic’s MIC against E. coli ATCC 25922. Positive correlation between cLog P values of antibiotics and values of sensitizing effects. I represents β-lactam antibiotics; II represents aminoglycosides antibiotics; III represents high molecular weight antibiotics; IV represents other antibiotics. PEN, penicillin G; AMP, ampicillin; CRO, Ceftriaxone Sodium; AZT, aztreonam; IMP, imipenem; TOB, tobramycin; AMK, amikacin; GEN, gentamycin; KAN, kanamycin; RFP, rifampicin; ERY, erythromycin; CLR, clarithromycin; CLI, clindamycin; TET, tetracycline; DOX, doxycycline; CHL, chloramphenicol; PMB, polymyxin B; DAP, daptomycin; Tava, tavaborole. A30, T30, K30 indicates the evolving E. coli ATCC 25922 strains collected at day 30 in the presence of sub-MIC concentration of tavaborole, tobramycin, and amikacin, respectively. At30 indicates the evolving strains collected at day 30 in the presence of sub-MIC concentration of tavaborole with addition of tobramycin. Each MIC measurement is repeated three times.** Figure S5.** Checkerboard graph of tavaborole combined with tobramycin or amikacin to kill induced drug-resistant bacterial strains. Black arrows indicate the point used to calculate FICI. All experiments are performed three times.** Figure S6.** Tavaborole LeuRS-tRNALeu cocrystal structure of editing active site. Best pose of the tavaborole-tRNALeu adduct, showing the interacting residues in three-dimension (A) and two-dimension format (B).** Figure S7.** Gene ontology (GO) annotation analysis of the differential expression proteins (DEPs) in E. coli ATCC 25922 treated with amikacin or amikacin-tavaborole combination. There are three parts including biological processes (A), molecular function (B), and cellular component (C). Each group had two replicates. An adjusted p-value < 0.05 (Fisher’s exact test).** Figure S8.** KEGG enrichment analysis of differential expression proteins (DEPs) in E. coli ATCC 25922 after exposure to amikacin or the combination of amikacin plus tavaborole. Each group had two replicates. An adjusted p-value < 0.05 (Fisher’s exact test).** Table S1.** Tavaborole interacts with LeuRS.

## Data Availability

The datasets used and/or analyzed during the current study are available from the corresponding author on reasonable request.

## Abbreviations

UTI: Urinary tract infection
AGAs: Aminoglycosides antibiotics
MDR: Multidrug-resistant
HTS: High Throughput Screen
RFP: Rifampicin
TOB: Tobramycin
AMK: Amikacin
PMB: Polymyxin B
DMSO: Dimethyl sulfoxide
FICs: Fractional inhibitory concentrations
FICI: FIC index
CFUs: Colony-forming units
CV: Crystal violet
MATH: Microbial adherence to hydrocarbon
RBCs: Red blood cells
HC_50_: The concentration of 50% hemolysis
H&E: Hematoxylin and eosin
OD: Optical density

## Availability of data and materials

The mass spectrometry proteomics data have been deposited to the ProteomeXchange Consortium via the PRIDE partner repository with the dataset identifier PXD033907”.
