# Supplementary material for: Drug synergy discovery of tavaborole and aminoglycosides against Escherichia coli using high throughput screening
Source: AMB Express. 2022 Dec 1;12:151. doi: 10.1186/s13568-022-01488-6 (PMC9715904; doi:10.1186/s13568-022-01488-6)
Supplement: Supplementary file 1 — Additional file 1: Figure S1. Chequerboard microdilution assays of AMK or TOB and tavaborole against E. coli Y0064, Y9395, Y9592, and Y9633 (all of them were XDR strains). Higher bacteria loading and lower growth-inhibition ability are represented by dark-red regions. X- and Y-axes were as log2 scale. The experiment was conducted with three biological replicates. Figure S2. Checkerboard dilution method of tavaborole combined with TOB against K. pneumoniae ATCC 700603, and with AMK against P. aeruginosa PAO1 and A. baumannii ATCC 19606. The experiment was conducted with three biological replicates. Synergy is defined as an FIC index of ≤ 0.5. Figure S3. Time-dependent growth or killing curves of E. coli ATCC 25922 treated with DMSO (Ctrl), tavaborole (Tava, 4 μg/ml) or sub-MIC of amikacin (AMK, 2 or 1 or 0.5 μg/ml) alone or in combination (Tava +AMK, 4 μg/ml + 2 μg/ml or 4 μg/ml + 1 μg/ml or 4 μg/ml + 0.5 μg/ml). The bacterial CFU/mL at specific time points during 24 h were determined. The experiment was performed with three biological replicates. Figure S4. Sensitizing effect is calculated by fold reduction of antibiotic’s MIC against E. coli ATCC 25922. Positive correlation between cLog P values of antibiotics and values of sensitizing effects. I represents β-lactam antibiotics; II represents aminoglycosides antibiotics; III represents high molecular weight antibiotics; IV represents other antibiotics. PEN, penicillin G; AMP, ampicillin; CRO, Ceftriaxone Sodium; AZT, aztreonam; IMP, imipenem; TOB, tobramycin; AMK, amikacin; GEN, gentamycin; KAN, kanamycin; RFP, rifampicin; ERY, erythromycin; CLR, clarithromycin; CLI, clindamycin; TET, tetracycline; DOX, doxycycline; CHL, chloramphenicol; PMB, polymyxin B; DAP, daptomycin; Tava, tavaborole. A30, T30, K30 indicates the evolving E. coli ATCC 25922 strains collected at day 30 in the presence of sub-MIC concentration of tavaborole, tobramycin, and amikacin, respectively. At30 indicates the evolving strains collected at [file 13568_2022_1488_MOESM1_ESM.docx]

**Supplement Methods**

**Post-antibiotic effects (PAE) *in vitro***

The analysis of *E.coli* ATCC 25922 was performed as described with minor modifications (Oh et al. 2019). The logarithmic phase cultures were adjusted to 0.5 McF and diluted 1:100 in MH medium with final concentration approximately 1.5×10^6^ CFU/ml. Exposed to tavaborole, tobramycin alone (1× to 4× MIC ) and their combination (4× MIC tavaborole plus 4× MIC tobramycin) for 1 h in 37 ℃ constant temperature shaker, the compounds were removed by 1:1000 dilution into 10 ml fresh MH medium and continued to culture for 12 h. 100 μl aliquots were removed for turbidity measurements, and ten-fold serially diluted suspensions were plated on blood agar plates for calculating CFUs following incubation at 37 °C for 24 h at the time points of 0, 2, 4, 8, 12, and 24 h.

**Post-antibiotic sub-MIC effects (PA-SME) *in vitro***

After PAE induction for 1 h with compounds as described above. 1:1000 dilution cultures were exposed into 1/4 × MIC tobramycin and further incubation with agitation for 12 h. Viability counts were performed at time point of 1, 2, 3, 4, 6, 12 h. The PA-SME was defined as *T_ps_ – C*, where *T_ps_* is the time required for cultures previously exposed to compounds and then exposed to subinhibitory concentrations to increase from 3 log10 to 4 log 10 above the counts immediately after thousand-fold dilution and *C* is the corresponding time for the antibiotic-free growth control not exposed to any compounds (Jacobs et al. 2003)

**Outer membrane permeability assay**

The bacterial outer membrane permeability assay induced by tavaborole was investigated using fluorescent probe 1-N-phenylnaphthylamine (NPN) as previously described (De Oliveira et al. 2020). Mid-log phase of *E.coli* ATCC 25922 cells were pelleted after centrifugation of 4,000 rpm for 10 min, washed three times with HEPES buffer (5 mM, pH 7.4) including glucose (5 mM) and diluted to a OD_630_ nm of 0.5. Subsequently, the bacterial suspensions were allocated to a 96-well black plate, and NPN (10 μM) was added and mixed with tavaborole or polymyxin B. The fluorescence was recorded at λ_ex_/ λ_em_ = 350/420 nm and utilized by a microplate reader until there was no further growth in signal.

**Cytoplasmic membrane depolarization assay**

The cytomembrane depolarization was measured by using 3,3-dipropylthiadi-carbocyanine iodide (DiSC_3_(5)) fluorescent dye as described previously with minor modifications (Liu et al. 2020a). In short, mid-log growth phase of *E.coli* ATCC 25922 was collected by centrifugation at 4,000 rpm for 10 min and washed twice by HEPES buffer (5 mM, pH 7.2) with 5 mM glucose and 100 mM KCl solution. And the bacterial suspension was resuspended in the buffer to the OD_630_ of 0.2, and then incubated with DiSC_3_(5) (final concentration of 2 μM) for 45 min in dark. Next, transfer the above mixture into a 96-well black plate in the presence of serially diluted concentrations of tavaborole. Fluorescent intensity changes were captured every 30 seconds for a total of 5 minutes by the microplate reader (λ_ex_=622 nm, λ_em_=670 nm). Polymyxin B was set as a positive control and DMSO as a negative control. The experiments were repeated by three times.

**Membrane fluidity assessment**

Membrane fluidity assessment was conducted as previously described with minor modification(Kim et al. 2019). Mid-log growth phase of *E.coli* ATCC 25922 was 1: 1000 diluted in fresh LB media and incubated to a OD_630_ of 0.5. The Laurdan liquid dye (6-dodecanoyl-2-(dimethylamino) naphthalene) was added with 10 μM of final concentration and incubated for 10 min at room temperature. Centrifuged at 4,000 rpm for 8 min, the supernatant was removed and the sediment was washed three times with 1×PBS. Treated with various concentration of tavaborole (1/2 × MIC to 2 × MIC), the fluorescence intensities of five-fold enrichment were captured at λ_ex_=350 and λ_em_= 440 nm and 490 nm. Ciprofloxacin or colistin as inversely positive control. The generalized polarization (GP) values of Laurdan were calculated as follows:

GP=$\frac{I_{440}-I_{490}}{I_{440}+I_{490}}$

**Intracellular ATP measurement**

The operation of detecting intracellular ATP levels of *E.coli* ATCC 25922 was followed with an instruction manual of Enhanced ATP Assay Kit (Beyotime, China) (Liu et al. 2020b). Overnight *E.coli* cultures were washed three times by 1 × PBS (pH 7.4) and resuspended to obtain an OD630 of 0.5. Treated with various concentration of tavaborole (1/2 × MIC to 2 × MIC) for 1 h, bacterial suspension was centrifuged at 12000 rpm for 5 min and the supernatant was removed. Afterwards, the bacterial sediment was lysed and centrifuged again with same parameters to obtain the supernatant for intracellular ATP levels measurement. 100 μl of 1:9 diluted detecting solution was added to 96-well plate and incubated at 25 °C for 3-5 min. After adding 20 μl/well prepared supernatant, the luminescence was captured by Multimode Plate Reader (PerkinElmer, USA). Intracellular ATP level was calculated in accordance with luminescence signals.

**Supplement Figures**


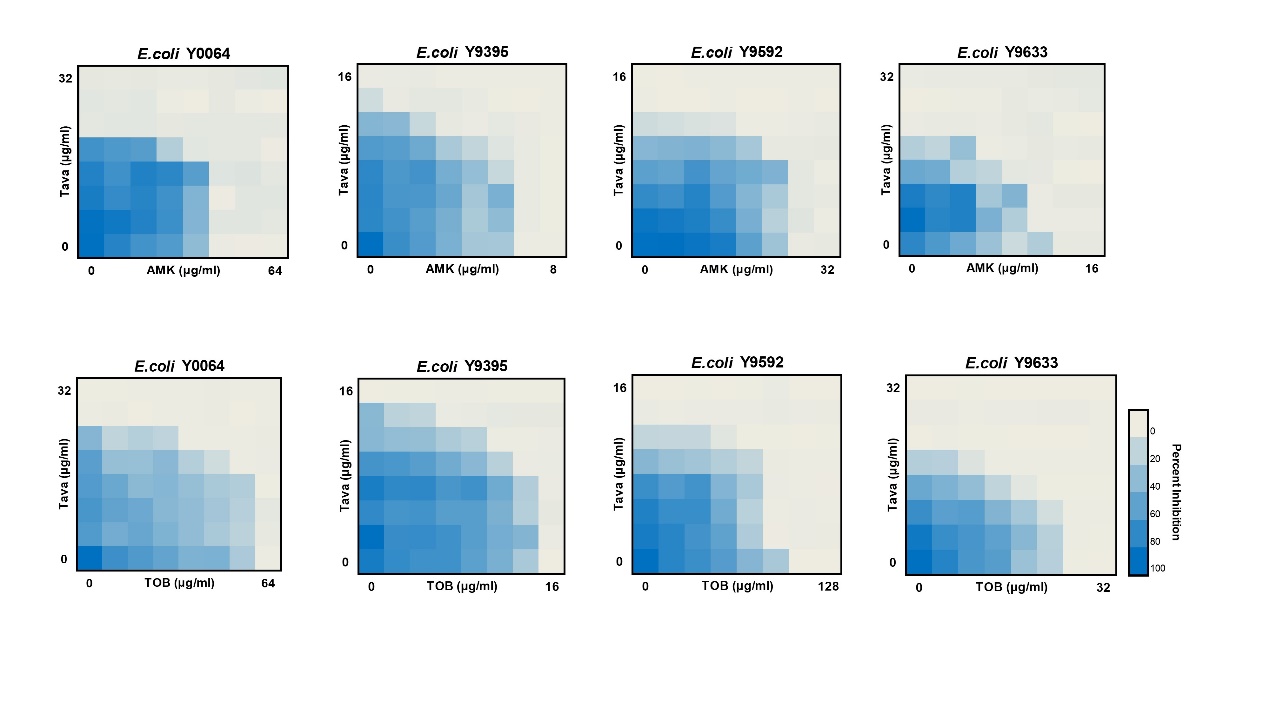


**Figure S1** Chequerboard microdilution assays of AMK or TOB and tavaborole against *E. coli* *Y0064*, *Y9395*, *Y9592*, and *Y9633* (all of them were XDR strains). Higher bacteria loading and lower growth-inhibition ability are represented by dark-red regions. X- and Y-axes were as log2 scale. The experiment was conducted with three biological replicates.


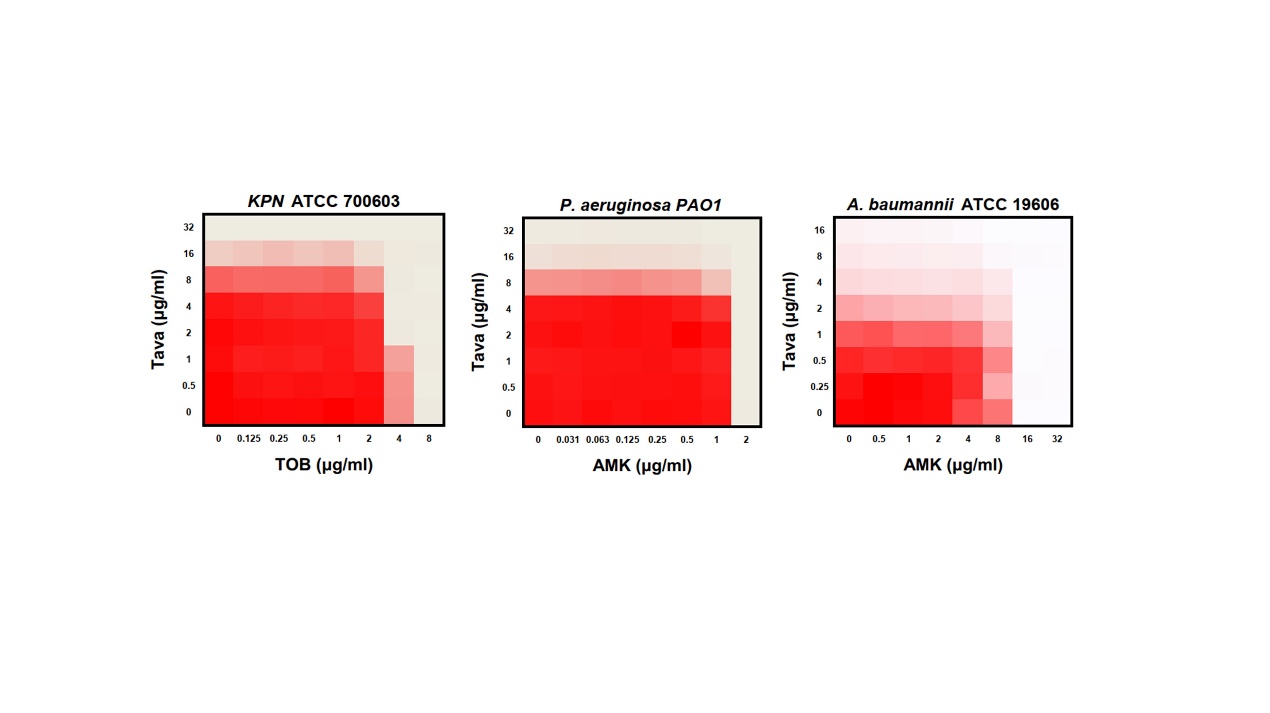


**Figure S2** Checkerboard dilution method of tavaborole combined with TOB against *K. pneumoniae* ATCC 700603, and with AMK against *P.* *aeruginosa* PAO1 and *A. baumannii* ATCC 19606. The experiment was conducted with three biological replicates. Synergy is defined as an FIC index of ≤ 0.5.


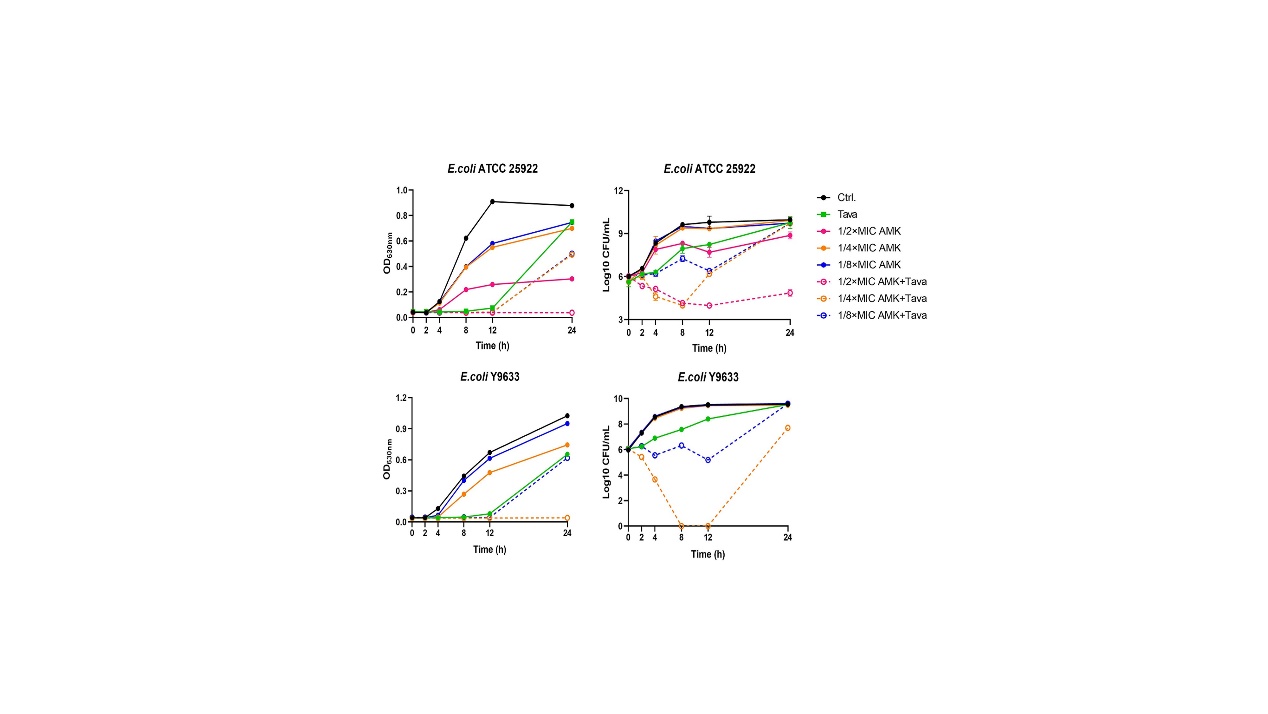


**Figure S3** Time-dependent growth or killing curves of *E. coli* ATCC 25922 treated with DMSO (Ctrl), tavaborole (Tava, 4 μg/ml) or sub-MIC of amikacin (AMK, 2 or 1 or 0.5 μg/ml) alone or in combination (Tava +AMK, 4 μg/ml + 2 μg/ml or 4 μg/ml + 1 μg/ml or 4 μg/ml + 0.5 μg/ml). The bacterial CFU/mL at specific time points during 24 h were determined. The experiment was performed with three biological replicates.


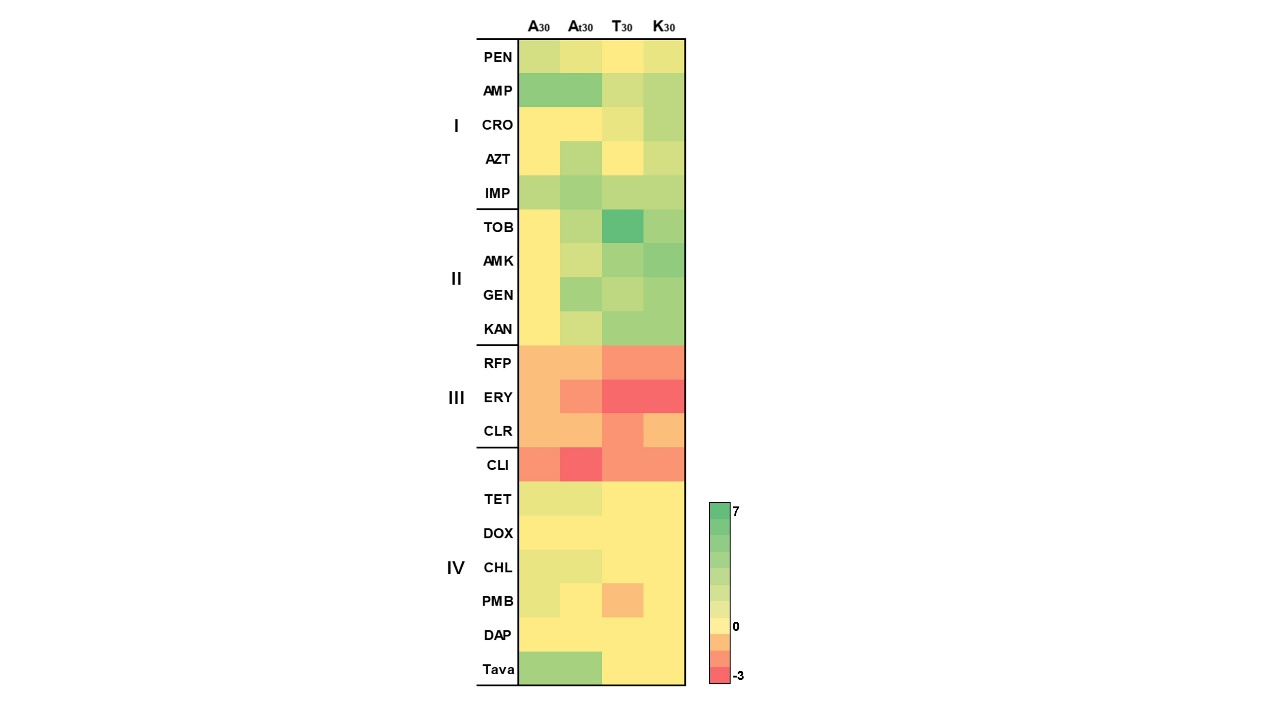


**Figure S4** Sensitizing effect is calculated by fold reduction of antibiotic’s MIC against *E. coli ATCC* 25922. Positive correlation between cLog P values of antibiotics and values of sensitizing effects.Ⅰrepresents β-lactam antibiotics; Ⅱrepresents aminoglycosides antibiotics; Ⅲ represents high molecular weight antibiotics; Ⅳ represents other antibiotics. PEN, penicillin G; AMP, ampicillin; CRO, Ceftriaxone Sodium; AZT, aztreonam; IMP, imipenem; TOB, tobramycin; AMK, amikacin; GEN, gentamycin; KAN, kanamycin; RFP, rifampicin; ERY, erythromycin; CLR, clarithromycin; CLI, clindamycin; TET, tetracycline; DOX, doxycycline; CHL, chloramphenicol; PMB, polymyxin B; DAP, daptomycin; Tava, tavaborole. A30, T30, K30 indicates the evolving *E. coli* ATCC 25922 strains collected at day 30 in the presence of sub-MIC concentration of tavaborole, tobramycin, and amikacin, respectively. A_t30_ indicates the evolving strains collected at day 30 in the presence of sub-MIC concentration of tavaborole with addition of tobramycin. Each MIC measurement is repeated three times.


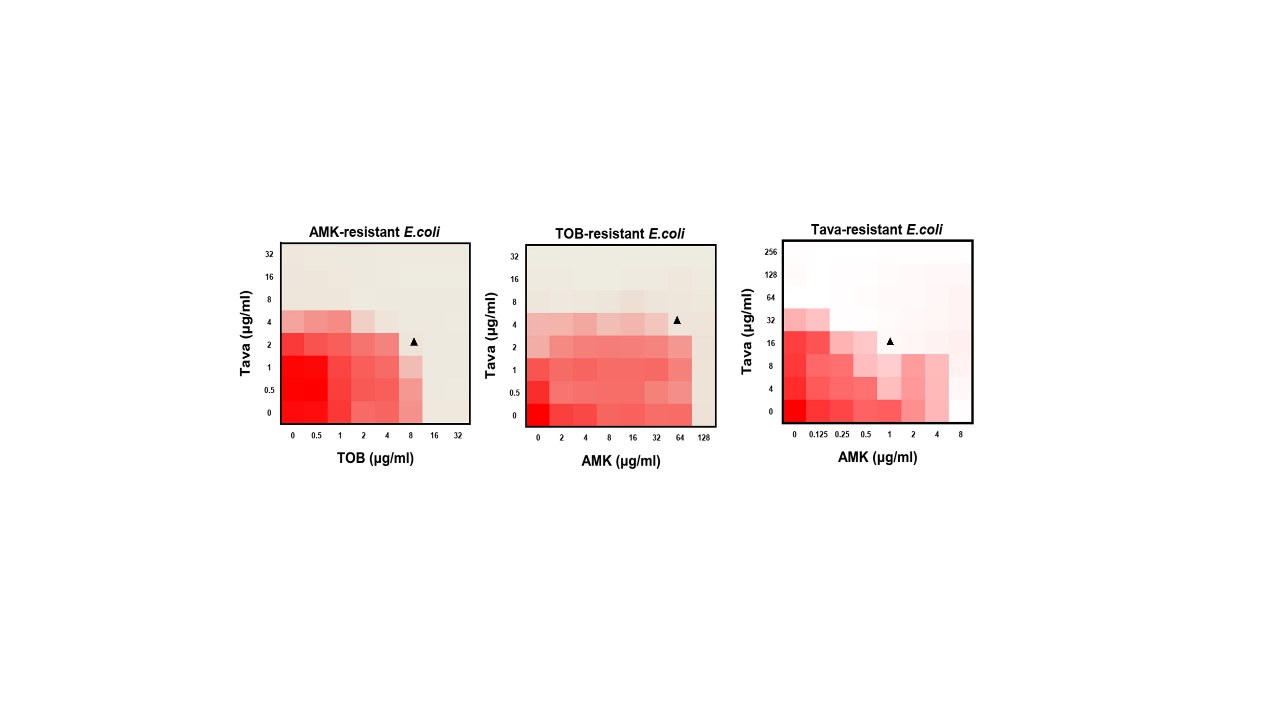


**Figure S5** Checkerboard graph of tavaborole combined with tobramycin or amikacin to kill induced drug-resistant bacterial strains. Black arrows indicate the point used to calculate FICI. All experiments are performed three times.


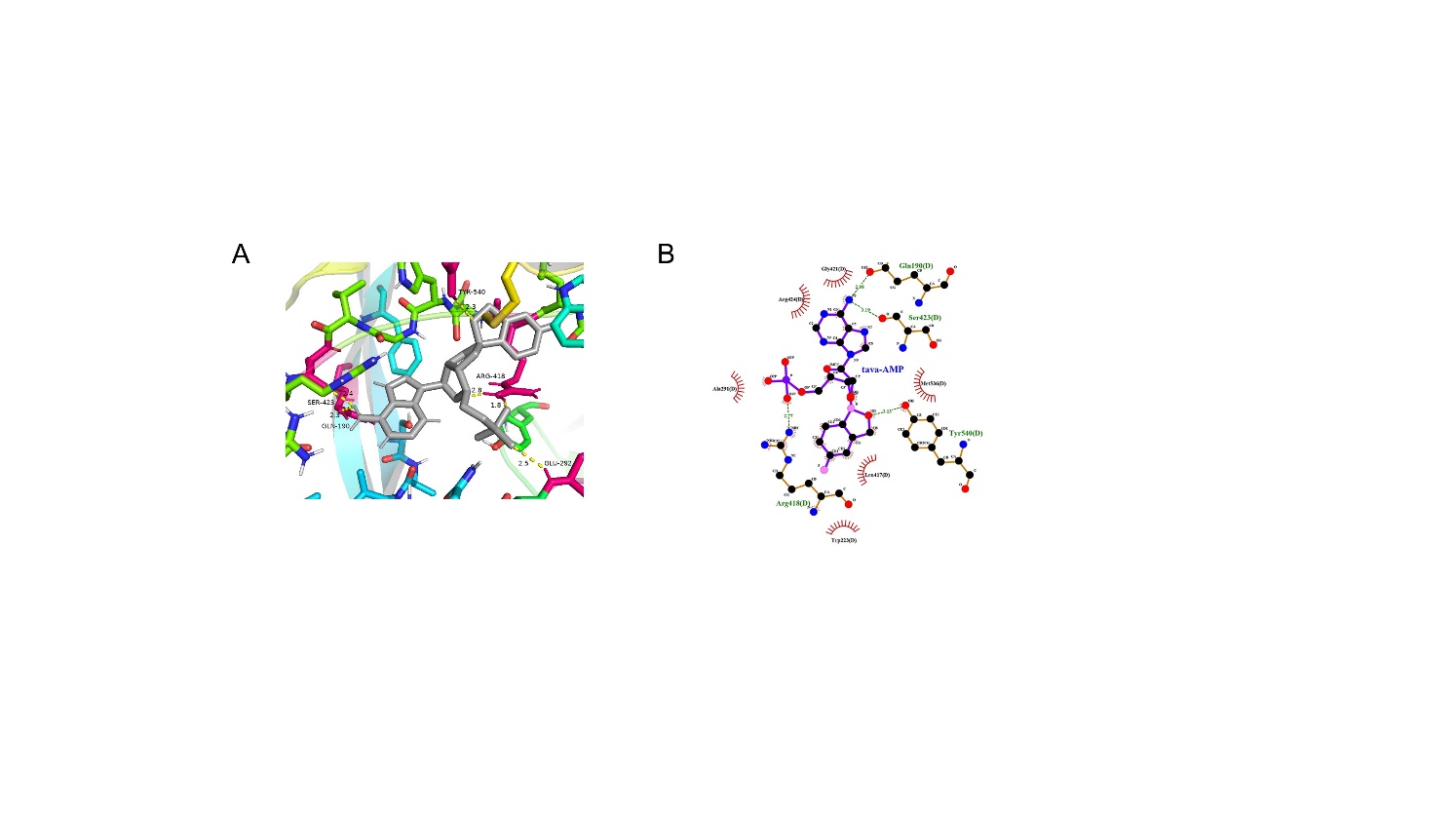


**Figure S6** Tavaborole LeuRS-tRNA^Leu^ cocrystal structure of editing active site. Best pose of the tavaborole-tRNA^Leu^ adduct, showing the interacting residues in three-dimension (A) and two-dimension format (B).


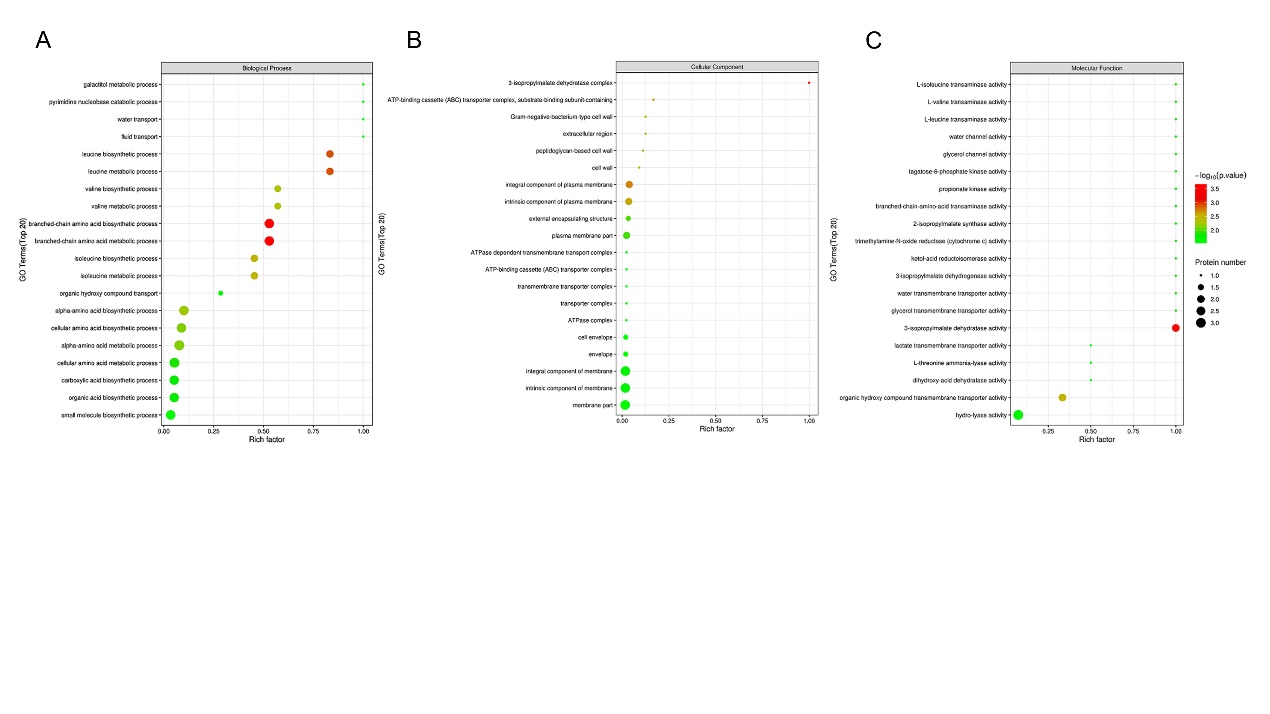


**Figure S7** Gene ontology (GO) annotation analysis of the differential expression proteins (DEPs) in *E. coli* ATCC 25922 treated with amikacin or amikacin-tavaborole combination. There are three parts including biological processes (A), molecular function (B), and cellular component (C). Each group had two replicates. An adjusted *p*-value < 0.05 (Fisher’s exact test).


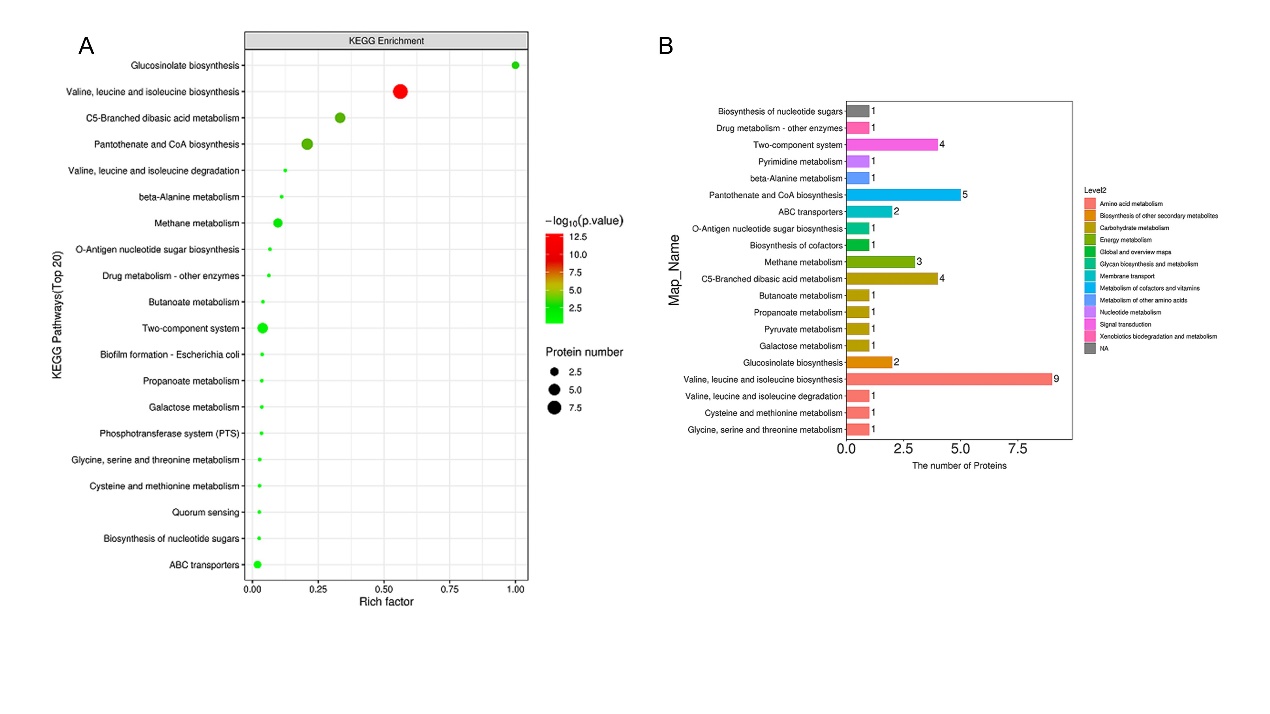
**Figure S8** KEGG enrichment analysis of differential expression proteins (DEPs) in *E. coli* ATCC 25922 after exposure to amikacin or the combination of amikacin plus tavaborole. Each group had two replicates. An adjusted *p*-value < 0.05 (Fisher’s exact test).

**Supplement Table 1** Tavaborole interacts with LeuRS

| Interaction Type | Residue | Amino Acid | Distance |
| --- | --- | --- | --- |
| Hydrophobic Interactions | 223D | TRP | 3.58 |
|  | 417D | LEU | 3.12 |
| Hydrogen Bonds | 168D | ASN | 3.30 |
|  | 190D | GLN | 3.41 |
|  | 292D | GLU | 2.53 |
|  | 418D | ARG | 2.77 |
|  | 423D | SERR | 2.36 |
|  | 424D | ARG | 2.6 |
|  | 540D | TYR | 2.27 |
| Halogen Bonds | 223D | TRP | 3.92 |
| Salt Bridges | 418D | ARG | 4.97 |

**Reference**

De Oliveira DMP, Bohlmann L, Conroy T, Jen FE, Everest-Dass A, Hansford KA, Bolisetti R, El-Deeb IM, Forde BM, Phan MD, Lacey JA, Tan A, Rivera-Hernandez T, Brouwer S, Keller N, Kidd TJ, Cork AJ, Bauer MJ, Cook GM, Davies MR, Beatson SA, Paterson DL, McEwan AG, Li J, Schembri MA, Blaskovich MAT, Jennings MP, McDevitt CA, von Itzstein M, Walker MJ (2020) Repurposing a neurodegenerative disease drug to treat Gram-negative antibiotic-resistant bacterial sepsis. Science translational medicine 12(570) doi:10.1126/scitranslmed.abb3791

Jacobs MR, Bajaksouzian S, Appelbaum PC (2003) Telithromycin post-antibiotic and post-antibiotic sub-MIC effects for 10 Gram-positive cocci. The Journal of antimicrobial chemotherapy 52(5):809-12 doi:10.1093/jac/dkg437

Kim W, Zou G, Hari TPA, Wilt IK, Zhu W, Galle N, Faizi HA, Hendricks GL, Tori K, Pan W, Huang X, Steele AD, Csatary EE, Dekarske MM, Rosen JL, Ribeiro NQ, Lee K, Port J, Fuchs BB, Vlahovska PM, Wuest WM, Gao H, Ausubel FM, Mylonakis E (2019) A selective membrane-targeting repurposed antibiotic with activity against persistent methicillin-resistant *Staphylococcus aureus*. Proceedings of the National Academy of Sciences of the United States of America 116(33):16529-16534 doi:10.1073/pnas.1904700116

Liu Y, Jia Y, Yang K, Li R, Xiao X, Zhu K, Wang Z (2020a) Metformin Restores Tetracyclines Susceptibility against Multidrug Resistant Bacteria. Advanced science (Weinheim, Baden-Wurttemberg, Germany) 7(12):1902227 doi:10.1002/advs.201902227

Liu Y, Jia Y, Yang K, Tong Z, Shi J, Li R, Xiao X, Ren W, Hardeland R, Reiter RJ, Wang Z (2020b) Melatonin overcomes MCR-mediated colistin resistance in Gram-negative pathogens. Theranostics 10(23):10697-10711 doi:10.7150/thno.45951

Oh JT, Cassino C, Schuch R (2019) Postantibiotic and Sub-MIC Effects of Exebacase (Lysin CF-301) Enhance Antimicrobial Activity against *Staphylococcus aureus*. Antimicrobial agents and chemotherapy 63(6) doi:10.1128/AAC.02616-18
